# Physiological changes in the regulation of calcium and phosphorus utilization that occur after the onset of egg production in commercial laying hens

**DOI:** 10.3389/fphys.2024.1465817

**Published:** 2024-09-25

**Authors:** R. Alejandra Garcia-Mejia, Micaela Sinclair-Black, Lyssa R. Blair, Roselina Angel, Bibiana Jaramillo, Prafulla Regmi, Nabin Neupane, Monika Proszkowiec-Weglarz, Xabier Arbe, David Cavero, Laura E. Ellestad

**Affiliations:** ^1^ Department of Poultry Science, University of Georgia, Athens, GA, United States; ^2^ Department of Animal and Avian Sciences, University of Maryland, College Park, MD, United States; ^3^ Iluma Alliance, Durham, NC, United States; ^4^ Animal Biosciences and Biotechnology Laboratory, United States Department of Agriculture-Agricultural Research Service, Beltsville, MD, United States; ^5^ H&N International, Cuxhaven, Germany

**Keywords:** vitamin D_3_, medullary bone, breaking strength, mineral homeostasis, shell gland, kidney, ileum

## Abstract

At the onset of egg production, physiological changes governing calcium and phosphorus utilization must occur to meet demands for medullary bone formation and eggshell mineralization. The objective of this study was to identify these changes and determine if they are influenced by dietary supplementation with 1α-hydroxycholecalciferol (AlphaD3^™^, Iluma Alliance). Commercial laying hens fed either a control or AlphaD3-supplemented diet beginning at 18 weeks of age were sampled at 18 (n = 8) and 31 weeks (n = 8/diet) to evaluate mRNA expression associated with calcium and phosphorus utilization in kidney, shell gland, ileum, and liver, circulating vitamin D_3_ metabolites, and bone quality parameters in humerus, tibia, and keel bone. Though diet did not heavily influence gene expression at 31 weeks, several significant differences were observed between 18- and 31-week-old hens. Heightened sensitivity to hormones regulating calcium and phosphorus homeostasis was observed at 31 weeks, indicated by increased parathyroid hormone receptor 1, calcium-sensing receptor, calcitonin receptor, and fibroblast growth factor 23 receptors in several tissues. Increased renal expression of 25-hydroxylase and vitamin D binding protein (***DBP***) at 31 weeks suggests kidney participates in local vitamin D_3_ 25-hydroxylation and DBP synthesis after egg production begins. Biologically active 1,25(OH)_2_D_3_ was higher at 31 weeks, with correspondingly lower inactive 24,25(OH)_2_D_3_. Increased expression of plasma membrane calcium ATPase 1 and calbindin in kidney, shell gland, and ileum suggests these are key facilitators of calcium uptake. Elevated renal inorganic phosphorus transporter 1 and 2 and sodium-dependent phosphate transporter IIa at 31 weeks suggests increased phosphorus excretion following hyperphosphatemia due to bone breakdown for eggshell formation. Diet did influence bone quality parameters. Bone mineral density in both humerus and tibia was higher in AlphaD3-supplemented hens at 31 weeks. Tibial bone mineral content increased between 18 and 31 weeks, with AlphaD3-supplemented hens increasing more than control hens. Moreover, control hens exhibited diminished tibial breaking strength at 31 weeks compared to hens at 18 weeks, while AlphaD3-supplemented hens did not. Together, these results indicate supplementation with AlphaD3 enhanced bone mineralization during the medullary bone formation period and elucidate the adaptive pathways regulating calcium and phosphorus utilization after the onset of lay.

## 1 Introduction

At the onset of egg production, commercial laying hens adapt physiological mechanisms that facilitate the utilization of calcium and phosphorus, primarily for the purpose of eggshell mineralization and medullary bone formation (Nys et al., 1986b; Wu et al., 1994). These adaptive changes begin to occur at the onset of sexual maturity between 17 and 18 weeks (Lei et al., 2020; Bahry et al., 2023) and involve specific organs important for absorption, transport, and utilization of calcium and phosphorus that are necessary for egg production (Bar, 2008). Described changes that occur at the onset of lay include increased intestinal mineral absorption (Nys et al., 1992a), renal mineral reabsorption and excretion (Wideman, 1987), calcium uptake and utilization by the shell gland (Corradino et al., 1993; Samiullah and Roberts, 2014), and medullary bone formation (Dacke et al., 1993; Kerschnitzki et al., 2014). This study provides a holistic physiological approach to understanding changes in the molecular mechanisms associated with calcium and phosphorus utilization before and after the onset of egg production.

Hormonal regulation of calcium and phosphorus homeostasis is influenced by multiple factors throughout the 24-h egg formation cycle (Sinclair-Black et al., 2023). Eggshell formation causes fluctuations in plasma ionized calcium levels (Sinclair-Black et al., 2024) that are detected by the plasma membrane protein calcium-sensing receptor (**CASR**) (Diaz et al., 1997; Hui et al., 2021). A decrease in calcium triggers the release of parathyroid hormone (**PTH**) that binds to parathyroid hormone receptor 1 (**PTH1R**) in target tissues (Pinheiro et al., 2012), stimulating renal calcium reabsorption and phosphorus excretion (Elaroussi et al., 1993), renal production of biologically active 1,25(OH)_2_D_3_ via 1α-hydroxylase (Fraser and Kodicek, 1973), intestinal calcium absorption (Nemere and Norman, 1986), and bone breakdown (Miller, 1978) releasing both calcium and phosphorus into the blood. As circulating calcium and phosphorus levels subsequently rise, calcitonin (**CALC)** opposes effects of PTH by acting through calcitonin receptor (**CALCR**), primarily by inhibiting bone remodeling (Warshawsky et al., 1980) and promoting renal calcium excretion (Cochran et al., 1970). Circulating levels of phosphorus are regulated by fibroblast growth factor 23 **(FGF23)**, a hormone produced by bone in response to hyperphosphatemia in chickens (Wang et al., 2018; Gloux et al., 2020a). Its action is mediated by fibroblast growth factor receptors 1–4 (**FGFR1, FGFR2, FGFR3,** and **FGFR4)** in conjunction with the co-receptor Klotho (**KL**). In mammals (Shimada et al., 2004b; Perwad et al., 2005) and chickens (Ren et al., 2017), FGF23 induces renal excretion of excess circulating phosphorus. It has also been shown to inhibit PTH secretion (Ben-Dov et al., 2007) and production of 1,25(OH)_2_D_3_ (Shimada et al., 2004a) in mammals. Many of the hormonal mechanisms regulating calcium and phosphorus homeostasis during egg formation are not fully understood, particularly the roles of CALC and FGF23 (Yasuoka et al., 1998; Ogawa et al., 2003; Sinclair-Black et al., 2023).

The hormonal form of vitamin D_3_, 1,25(OH)_2_D_3_, plays a major role in regulating calcium and phosphorus homeostasis. It can induce transcription of membrane mineral transporters and intracellular chaperone proteins involved in calcium and phosphorus absorption and utilization in intestine and kidney (Bar et al., 1990). To become active, dietary vitamin D_3_ is first constitutively hydroxylated by hepatic 25-hydroxylase to produce 25(OH)D_3_ (Tucker et al., 1973). These enzymes are encoded by the *CYP2R1* (Watanabe et al., 2013) or *CYP27A1* (Shang et al., 2023) genes. A second, rate-limiting hydroxylation is catalyzed by renal 1α-hydroxylase to produce biologically active 1,25(OH)_2_D_3_. This enzyme is encoded by *CYP27B1* in mammals and fish (Monkawa et al., 1997; Shinki et al., 1997; Chun et al., 2014). However, this gene has not been identified in chickens to date, despite prior publications reporting measurements in its expression as reviewed in Sinclair-Black et al. (2023). Commercially available 1α-hydroxycholecalciferol is a pre-hydroxylated vitamin D_3_ supplement (AlphaD3™, Iluma Alliance), requiring only the constitutive 25-hydroxylation referenced above. This could potentially improve the efficiency with which 1,25(OH)_2_D_3_ is produced. The inactive vitamin D_3_ metabolite, 24,25(OH)_2_D_3_, is generated through an alternative hydroxylation step carried out by 24-hydroxylase, encoded by *CYP24A1* (Armbrecht et al., 1997; Nemere, 1999).

The period between medullary bone formation that begins at the onset of sexual maturity around 18 weeks of age (Hudson et al., 1993; Bahry et al., 2023) and peak egg production around 31 weeks of age assumes significance, as hens must develop and sustain the metabolic capacity to meet high mineral requirements throughout their productive life. Therefore, this study sought to elucidate physiological changes associated with calcium and phosphorus uptake and utilization that occur during the onset of lay between 18 and 31 weeks of age and determine if supplementation with AlphaD3 influenced these changes. The objectives were to measure 1) expression profiles of genes for hormone receptors mediating calcium and phosphorus homeostasis, enzymes involved in vitamin D_3_ metabolism, and mineral transporters in kidney, shell gland, ileum, and liver; 2) circulating vitamin D_3_ metabolites; and 3) parameters associated with skeletal integrity in hens fed control (18 and 31 weeks) or AlphaD3-supplemented (31 weeks) diets.

## 2 Materials and methods

### 2.1 Animals and experimental design

All animal procedures were approved by The Institutional Animal Care and Use Committee at the University of Georgia. The commercial strain Nick Chick white-egg laying hens (H&N International, Cuxhaven, Germany) used in this experiment were part of a larger flock reared at the University of Georgia’s Poultry Research Center according to the primary breeder’s nutritional and management guidelines (H&N International, 2020). In brief, chicks were raised on the floor from day of hatch through 16 weeks of age, when they were transferred to individual layer cages in an environmentally-controlled poultry house. Beginning at 18 weeks of age, light intensity was increased from 0.6 to 1.6 foot-candle, and the photoperiod was increased by 1 h of light per week until a 16L:8D program was achieved at 24 weeks of age. All birds had free access to water and were fed *ad libitum* with corn-soybean meal-based diets formulated to meet requirements of high-producing laying hens (Tables 1 and 2).

**TABLE 1:** Ingredients and nutrient composition of starter, grower, and developer diets.^a^.

Ingredients, as-fed %	Starter	Grower	Developer
Corn	61.986	58.337	56.787
Soybean meal, 47%	25.937	21.237	16.500
Wheat bran	-	8.500	-
Fish meal, 60%	2.000	-	-
Alfalfa meal	-	2.364	4.200
Wheat middlings	5.000	5.000	16.600
Fine limestone	1.460	1.688	1.500
Soybean oil	1.000	1.000	0.800
Mono-dicalcium phosphate, 21%	0.767	0.320	0.080
Arbocel^®^ ^b^	-	0.500	1.111
Vitamin premix	0.500^c^	0.050^d^	0.050^d^
Sand	0.400	-	1.250
Sodium chloride	0.290	0.270	0.260
DL Methionine	0.187	0.151	0.129
L-Lysine HCL	0.147	0.108	0.111
Coban 90	0.090	0.090	0.090
Trace mineral premix^e^	0.080	0.080	0.080
Sodium bicarbonate	0.078	0.155	0.310
Choline chloride, 60%	-	0.073	0.040
Bacitracin methylene disalicylate (BMD)^f^	0.050	0.050	0.050
L-Threonine	0.023	0.022	0.025
L-Tryptophan	-	-	0.012
Axtra^®^ PHY GOLD^g^	0.005	0.005	0.005
Formulated nutrient composition (analyzed)			
Crude Protein	19.185 (17.700)	17.700 (16.600)	15.900 (16.200)
Crude fiber	1.942 (2.400)	3.500 (4.000)	4.219 (4.600)
Calcium	1.050 (0.890)	1.000 (0.840)	1.000 (0.720)
Phosphorus	0.601 (0.660)	0.559 (0.620)	0.480 (0.500)

^a^ The diets were fed as follows: starter (0–5 weeks of age), grower (6–10 weeks of age), developer (11–17 weeks of age). Hens sampled at 18 weeks (Baseline) continued to be fed developer diet through18 weeks.

^b^ JRS, Pharma LP, patterson, NY.

^c^ Provided the following per kilogram of diet: vitamin A, 11,022 IU; vitamin D_3_, 2204 IU; vitamin E, 22.05 IU; vitamin B12, 0.02 mg; Menadione, 2.21 mg; riboflavin, 8.82 mg; d-pantothenic acid, 22.05 mg; thiamine, 4.41 mg; niacin, 88.19 mg; vitamin B6, 4.41 mg; folic acid, 1.10 mg; choline, 382 mg; biotin, 0.40 mg

^d^ Provided the following per kilogram of diet: vitamin A, 10,019 IU; vitamin D_3_, 4519 IU; vitamin E, 41.34 IU; vitamin B12, 0.02 mg; Menadione, 2.48 mg; riboflavin, 13.78 mg; d-pantothenic acid, 19.29 mg; thiamine, 3.44 mg; niacin, 55.11 mg; vitamin B6, 4.82 mg; folic acid, 1.65 mg; biotin, 0.55 mg.

^e^ Provided the following per kilogram of diet: Calcium min, 25.6 mg; Calcium max, 33.6 mg; Manganese min, 107.2 mg; Zinc, 85.6 mg; Magnesium, 19.8 mg; Iron, 21 mg; Copper, 3.2 mg; Iodine, 0.8 mg; Selenium min, 0.3 mg.

^f^ Zoetis, Parsipanny, NJ.

^g^ Danisco Animal Nutrition & Health (IFF), cedar rapids, IA.

**TABLE 2:** Ingredients and nutrient composition of basal onset and layer diets.^a^.

Ingredients, as-fed %	Onset	Layer
Corn	49.444	45.923
Soybean meal, 47%	25.703	25.343
Wheat middlings	9.166	14.028
Coarse limestone	6.527	6.130
Fine limestone	2.798	3.306
Soybean oil	2.014	3.395
Alfalfa meal	2.000	-
Mono-dicalcium phosphate, 21%	1.358	0.770
Sodium chloride	0.300	0.260
DL Methionine	0.261	0.247
Sodium bicarbonate	0.116	0.128
Trace mineral premix^b^	0.080	0.076
L-Lysine HCL	0.045	0.028
Bacitracin methylene disalicylate (BMD)^c^	0.050	0.048
Arbocel^®^ ^d^	0.050	0.238
Choline chloride 60%	0.040	0.038
Vitamin premix^e^ ^,^ ^f^	0.025	0.025
L-Threonine	0.013	0.011
L-Tryptophan	0.010	0.009
Calculated nutrient content (analyzed)		
Crude Protein	17.890 (18.400)	17.203 (16.600)
Crude fiber	2.677 (4.000)	2.000 (4.100)
Calcium	4.000 (3.600)	3.990 (3.800)
Phosphorus	0.686 (0.730)	0.643 (0.550)

^a^ Basal onset and layer diets were mixed with all ingredients except the vitamin premix, split in half, and remixed with either the control (no Alpha D3) or the AlphaD3-supplemented premix. Diets were fed as follows: onset (17–22 weeks), layer (22–31 weeks). The AlphaD3-supplemented diet had 3.5 µg 1α-hydroxycholecalciferol/kg. Analyzed 1α-hydroxycholecalciferol level was below detection limit for the control diet and 2.67 and 3.05 μg/kg for the AlphaD3 onset and layer diets, respectively.

^b^ Provided the following per kilogram of diet: Calcium min, 25.6 mg; Calcium max, 33.6 mg; Manganese min, 107.2 mg; Zinc, 85.6 mg; Magnesium, 19.8 mg; Iron, 21 mg; Copper, 3.2 mg; Iodine, 0.8 mg; Selenium min, 0.3 mg.

^c^ Zoetis, Parsipanny, NJ.

^d^ JRS, Pharma LP, patterson, NY.

^e^ Control premix provided the following per kilogram of diet: vitamin A, 10,000 IU; vitamin D_3_ 2000 IU, Vitamin B-12, 30 mg; vitamin E, 3 IU; vitamin K (menadione), 3.5 mg; riboflavin, 10 mg; pantothenic acid, 45 mg; thiamine, 20 mg; niacin, 4 mg; pyridoxine, 0.006 mg; folic acid, 0.03 mg; biotin, 1 mg.

^f^ AlphaD3 premix provided the following per kilogram of diet: vitamin A, 10,000 IU; vitamin D_3_ 2000 IU, Vitamin B-12, 30 mg; vitamin E, 3 IU; vitamin K (menadione), 3.5 mg; riboflavin, 10 mg; pantothenic acid, 45 mg; thiamine, 20 mg; niacin, 4 mg; pyridoxine, 0.006 mg; folic acid, 0.03 mg; biotin, 1 mg; 1α-hydroxycholecalciferol, 3.5 µg.

At 17 weeks of age, 24 hens were randomly allocated to one of three different experimental groups (n = 8 hens/group): (1) Baseline, which continued to be fed the developer diet and were sampled at 18 weeks of age prior to photostimulation and the onset of lay; (2) control, which were fed basal onset (17–22 weeks) and layer (22–31 weeks) diets and sampled at 31 weeks of age; and (3) AlphaD3, which were fed basal onset (17–22 weeks) and layer (22–31 weeks) diets supplemented with AlphaD3™ [1α-hydroxycholecalciferol (Iluma Alliance, Durham, NC)] and sampled at 31 weeks of age. Each hen was considered an experimental unit, so there were 8 biological replicates for all parameters measured. To make the experimental onset and layer diets, basal diets were mixed with all ingredients except the vitamin premix, split in half, and then re-mixed with one of two different vitamin pre-mixes for control (no AlphaD3) and AlphaD3 (3.5 μg/kg of feed) treatments. Vitamin D_3_ inclusion for both diets was 2000 UI/kg, and AlphaD3 was supplemented on top of it.

### 2.2 Sample collection

Blood and tissues were collected from hens at 18 and 31 weeks of age. Hens sampled at 31 weeks of age were individually monitored, and blood and tissues were collected at 21 h post-oviposition when a hard-shelled egg was present in the shell gland to minimize variation associated with the daily laying cycle (Sinclair-Black et al., 2024). Whole blood (∼4.5 mL) was collected from the brachial vein into S-monovette collection tubes containing lithium heparin (SARSTEDT, Inc., Newton, NC), centrifuged at 1,500 x *g* and 4°C for 15 min to isolate plasma, and plasma was stored at −20°C until analyzed for vitamin D_3_ metabolites. Immediately following blood collection, hens were euthanized by intravenous administration of 1-mL pentobarbital sodium (Euthasol^®^, Virbac, Westlake, TX) into the brachial or medial metatarsal vein. The right lobe of the liver, central shell gland, caudal lobe of the right kidney (approximately 500 mg per tissue), and homogenized mucosal scrapings from the proximal 1/3 of the ileum (approximately 30 mg) were collected, immediately snap-frozen in liquid nitrogen, and stored at −80°C prior to total RNA isolation. Keel bone and right and left humerus and tibia were excised from each bird, cleaned of most of the muscle and connective tissue, and stored at −20°C until used for determination of bone parameters.

### 2.3 Vitamin D_3_ metabolite determination

The vitamin D_3_ metabolites 25-hydroxycholecalciferol, 24,25-dihydroxycholecalciferol, and 1,25-dihydroxycholecalciferol were analyzed by liquid chromatography-tandem mass spectrometry (Heartland Assays, Ames, IA). Each sample was analyzed in duplicate, and the average values of these duplicates were used for further analysis. The lower limits of detection (**LOD**) and quantification (**LOQ**) for each analyte are as follows: 25-hydroxycholecalciferol – 0.5 ng/mL (LOD) and 1.5 ng/mL (LOQ); 24,25-dihydroxycholecalciferol – 0.1 ng/mL (LOD) and 0.3 ng/mL (LOQ); and 1,25-dihydroxycholeaclcierol – 5.0 pg/mL (LOD) and 10 pg/mL (LOQ).

### 2.4 Total RNA isolation

Total RNA was isolated from approximately 30 mg of each tissue that was homogenized in QIAzol lysis reagent (Qiagen, Valencia, CA) following the manufacturer’s instructions. Tissues were homogenized using a Mini-BeadBeater (Biospec Products, Bartlesville, OK) in bursts of 45 s and rested on ice between bursts, with total homogenization times of 90 s for kidney, ileum, and liver and 145 s for shell gland. Precipitated total RNA was reconstituted using 200 µL nuclease-free water. Total RNA quantification was determined using a Nanodrop ND1000 spectrophotometer (ThermoFisher Scientific, Waltham, MA). Evaluation of RNA integrity was performed via agarose gel electrophoresis using a UV imaging system (BioSpectrum, Upland, CA) and visualized with the Visionworks LS software (Wasserburg, Germany).

### 2.5 Reverse transcription-quantitative polymerase chain reaction (RT-qPCR)

One μg total RNA was reverse transcribed into cDNA using M-MuLV reverse transcriptase (200 U; New England Biolabs, Ipswich, MA), RNaseOUT inhibitor (8U; Invitrogen, Carlsbad, CA), and a mixture of anchored oligo-dT (TTTTTTTTTTTTTTTTTTTTVN, Integrated DNA technologies, Coralville, IA) and random hexamer (ThermoFisher Scientific, Waltham, MA) primers in 20 µL reactions. As a control for genomic DNA contamination, equivalent amounts of total RNA from all samples were used to make a 1 µg pool that was then used to conduct a reaction with all components except the reverse transcriptase enzyme (**no RT**). The samples and no RT control were initially incubated with primers and dNTPs at 65°C for 5 min, then rested on ice for 1 min before addition of M-MuLV enzyme (except for the no RT reaction), M-MuLV buffer, and RNaseOUT. All samples and the no RT control were then incubated under the following conditions: 5 min at 25°C, 60 min at 42°C, and 20 min at 65°C. Each cDNA sample was diluted 10-fold upon the reaction’s completion prior to analysis by qPCR. The cDNA used for 18s ribosomal RNA amplification was further diluted up to 1:500. Intron-spanning primers (Table 3; Integrated DNA Technologies) for each transcript were designed using Primer3 plus Software (Untergasser et al., 2012). Quantitative PCR thermal cycling was conducted using a StepOne Real-Time PCR System (Applied Biosystems, Foster City, CA). Duplicate reactions for each sample (10 µL) were run for all genes, with each reaction containing 2 µL of template cDNA, 5 µL 2X PowerUp™ SYBR Green Master Mix (ThermoFisher), and 400 nM of each primer. The average Ct value of duplicate reactions was used for further analysis. Target genes were normalized to glyceraldehyde-3-phosphate dehydrogenase (***GAPDH***) in kidney and ileum, *18S* ribosomal RNA in shell gland, and cyclophilin (***CYCLO***) in liver. These genes were demonstrated to be the most stable out of all the ones evaluated and were not affected by age or diet. To normalize and transform the data, the following equations were used: ΔCt = Ct_Target gene_ – (Ct_GAPDH_ or Ct_18S_ or Ct_CYCLO_) and 2^−ΔCt^ (Ellestad et al., 2009; Vaccaro et al., 2022). Expression levels of each gene were then calculated relative to the average value for that gene at 18 weeks (Baseline) using equation (2^−ΔCt^)_target_/(average 2^−ΔCt^)_Baseline_, making the value for 18 weeks (Baseline) equal to 1 in all cases.

**TABLE 3:** Primers used for reverse transcription-quantitative PCR.

Gene symbol	Forward primer (5' -3')	Reverse primer (5' -3')	Transcript ID^a^
Hormonal signaling			
*Calcium homeostasis*			
*CASR*	CTG​CTT​CGA​GTG​TGT​GGA​CT	GAT​GCA​GGA​TGT​GTG​GTT​CT	55,986
*PTH1R*	CCA​AGC​TAC​GGG​AAA​CAA​AT	ATG​GCA​TAG​CCA​TGA​AAA​CA	08,796
*CALCR*	GCA​GTT​GCA​AGA​GCC​AAA​TA	AGC​TTT​GTC​ACC​AAC​ACT​CG	15,478
*Phosphorus homeostasis*			
*FGFR1*	GAC​AGA​CTT​CAA​CAG​CCA​GC	CCA​ACA​TCA​CAC​CCG​AGT​TC	66,938
*FGFR2*	CAG​GGG​TCT​CGG​AAT​ATG​AA	GCT​TCA​GCC​ATC​ACC​ACT​T	38,732
*FGFR3*	GGA​GTA​CTT​GGC​GTC​ACA​GA	TCT​AGC​AAG​GCC​AAA​ATC​AG	01,712
*FGFR4*	CTTGCCCGTCAAGTGGAT	TGA​AGA​TCT​CCC​ACA​TCA​GAA	25,332
*KL* ^b^	CCA​AGA​GAG​ATG​ATG​CCA​AA	CAT​CCA​GAA​GGG​ACC​AGA​CT	15,785^b^
Vitamin D_3_ metabolism and action			
*Vitamin D*_*3*_ *hydroxylation*			
*CYP2R1*	GGA​CAG​CAA​TGG​ACA​GTT​TG	AGG​AAA​ACG​CAG​GTG​AAA​TC	09,745
*CYP27A1*	CTG​TTA​TCA​AGG​AGA​CGC​TGA	TTG​GGG​AAG​AGG​TAG​TCT​CC	03,899
*CYP24A1*	TGG​TGA​CAC​CTG​TGG​AAC​TT	CTC​CTG​AGG​GTT​TGC​AGA​GT	59,161
*Vitamin D*_*3*_ *signaling*			
*DBP*	GGA​ACT​TCC​TCT​CCA​TGG​TC	AGC​AAG​CTC​TGT​TCG​ACA​TC	18,962
*VDR*	CTG​CAA​AAT​CAC​CAA​GGA​CA	CAT​CTC​ACG​CTT​CCT​CTG​C	96,121
*RXRA*	ACT​GCC​GCT​ACC​AGA​AGT​GT	GAC​TCC​ACC​TCG​TTC​TCG​TT	59,924
*RXRG*	GAA​GCC​TAC​ACG​AAG​CAG​AA	CCG​ATC​AGC​TTG​AAG​AAG​AA	49,224
Mineral uptake and utilization			
*Calcium transport*			
*CALB1*	AAG​CAG​ATT​GAA​GAC​TCA​AAG​C	CTG​GCC​AGT​TCA​GTA​AGC​TC	74,265
*SLC8A1*	TCA​CTG​CAG​TCG​TGT​TTG​TG	AAG​AAA​ACG​TTC​ACG​GCA​TT	13,920
*ATP2B1*	TTA​ATG​CCC​GGA​AAA​TTC​AC	TCC​ACC​AAA​CTG​CAC​GAT​AA	80,355
*TRPV6*	TAT​GCT​GGA​ACG​AAA​ACT​GC	TTG​TGC​TTG​TTG​GGA​TCA​AT	23,779
*Bicarbonate synthesis and transport*			
*CA2*	CCT​GAC​TAC​TCC​ACC​ACT​GC	TCT​CAG​CAC​TGA​AGC​AAA​GG	52,439
*SLC26A9*	TCC​ACG​ATG​CTG​TTT​TGT​TT	GAG​CTG​CTT​TCA​TCC​ACA​GA	01,070
*Inorganic phosphorus transport*			
*SLC20A1*	TAT​CCT​CCT​CAT​TTC​GGC​GG	CTC​TTC​TCC​ATC​AGC​GGA​CT	94,781
*SLC20A2*	CCA​TCC​CCG​TGT​ACC​TTA​TG	AGA​CAT​GGC​CAT​CAC​TCC​TC	51,992
*SLC34A1*	AAG​CCA​TCC​AGA​AGG​TCA​TC	CAG​TGG​TGT​GAT​GGC​TGA​G	58,978
*SLC34A2*	AAAGTGACGTGGACCATG	GAG​ACC​GAT​GGC​AAG​ATC​AG	23,222
Reference genes			
*GAPDH*	AGC​CAT​TCC​TCC​ACC​TTT​GAT	AGT​CCA​CAA​CAC​GGT​TGC​TGT​AT	23,323
*18S* ^c^	AGC​CTG​CGG​CTT​AAT​TTG​AC	CAA​CTA​AGA​ACG​GCC​ATG​CA	
*CYCLO*	GCT​CAT​GAA​ATT​GCC​CCT​GA	GCG​TAA​GCT​GCC​TTC​TCT​TTC​TC	15,382

^a^ Transcript identification from Ensembl chicken genome assembly GRCg6a http://www.ensembl.org/Gallus_gallus/Info/Index) preceded by ENSGALT000000 for all genes except *KL* and *18S* rRNA.

^b^ Transcript identification from Ensembl chicken genome assembly GRCg7b preceded by ENSGALT000100.

c Sequence of 18S rRNA, is not on the assembled chicken genome, and primers were designed based on the sequence in GenBank (Accession Number AF173612).

### 2.6 Bone parameters

Bone mineral density (**BMD**; g/cm^3^) and bone mineral content (**BMC**; g) were determined for keel, left humerus, and left tibia using a Lunar Prodigy dual-X-ray absorptiometry (**DEXA**) scanner (GE Healthcare, Chicago, IL). The orientation of the respective bones was the same in each scan for consistency. Bone-breaking strength was analyzed with a three-point bending test on the right tibia using a TA. HDplus Texture Analyser (Stable Micro Systems, Godalming, United Kingdom). Bones were thawed at room temperature, cleaned of any remaining surrounding soft tissue, wrapped in a damp paper towel to maintain moisture, and stored in a plastic bag at 4°C for 24 h before breaking. Each bone was positioned in the same orientation on two support points with a span of 40 mm between them. A load of 25 kg with a test speed of 1 mm/s was applied to the midpoint section of the same plane of each bone. The peak force required to break the bone was obtained from the deformation curve to determine breaking strength (**N**), and the total energy required for fracture **(N.mm)** was determined. Cortical thickness was determined from a straight cut made halfway between the midpoint and the distal end of the tibia and measured at the anterior, posterior, medial, and lateral positions using a Small Point Jaw Digital Caliper (INSIZE, Loganville, GA) that has a resolution of 0.001 mm and accuracy of ±0.03 mm.

Keel deviation prevalence, keel deviation severity, and presence of keel fractures were determined. Presence of keel bone deviation and deviation severity were measured from images taken of the ventral side of the keel bone using the image processing software Fiji (Schindelin et al., 2012). A straight line was drawn from the carina apex to the caudal tip of the keel bone, and distance (cm) for each lateral deviation from the straight line was recorded. To score the presence or absence of deviations, keels without any deviation (<0.25 cm) were scored as “0” and those with a deviation (>0.25 cm) were scored as “1.” For deviation severity, the maximum deviation distance was recorded and assessed with a 3-point scoring system adapted from that described in Heerkens et al. (2016): “0” for no deviations (<0.25 cm), “1” for mild deviations (0.25–0.75 cm), and “2” for severe deviations (>0.75 cm). To determine the presence of keel bone fracture, hens without any fractures were scored as “0” and hens with one or more fractures were scored as “1.” Each variable was analyzed independently.

### 2.7 Statistical analysis

All data were analyzed with JMP Pro 16 software (SAS Institute, Cary, NC), with hen as the experimental unit (n = 8 hens/group). The Shapiro-Wilk normality test was used to determine normal distribution of all the data. Gene expression, vitamin D_3_ metabolites, BMD, BMC, AUC, and cortical thickness were analyzed using a one-way analysis of variance (**ANOVA**), with experimental group as the model effect. Whenever the ANOVA indicated statistical significance (*p* ≤ 0.05), *post hoc* means comparisons were performed using Fisher’s test of least significant difference (**LSD**). A log transformation was applied to AUC data prior to analysis to achieve normality. Tibia breaking force was analyzed using Kruskal–Wallis test for non-parametric variables, and means comparison was performed using Fisher’s LSD test. For keel bone deviation and fracture scoring, the Chi-square probability test was performed for the effect of experimental group. For all variables analyzed, a power level between 75%–100% was achieved and differences were considered significant at *p* ≤ 0.05.

## 3 Results

### 3.1 Hormonal signaling

#### 3.1.1 Calcium homeostasis

**FIGURE 1:**
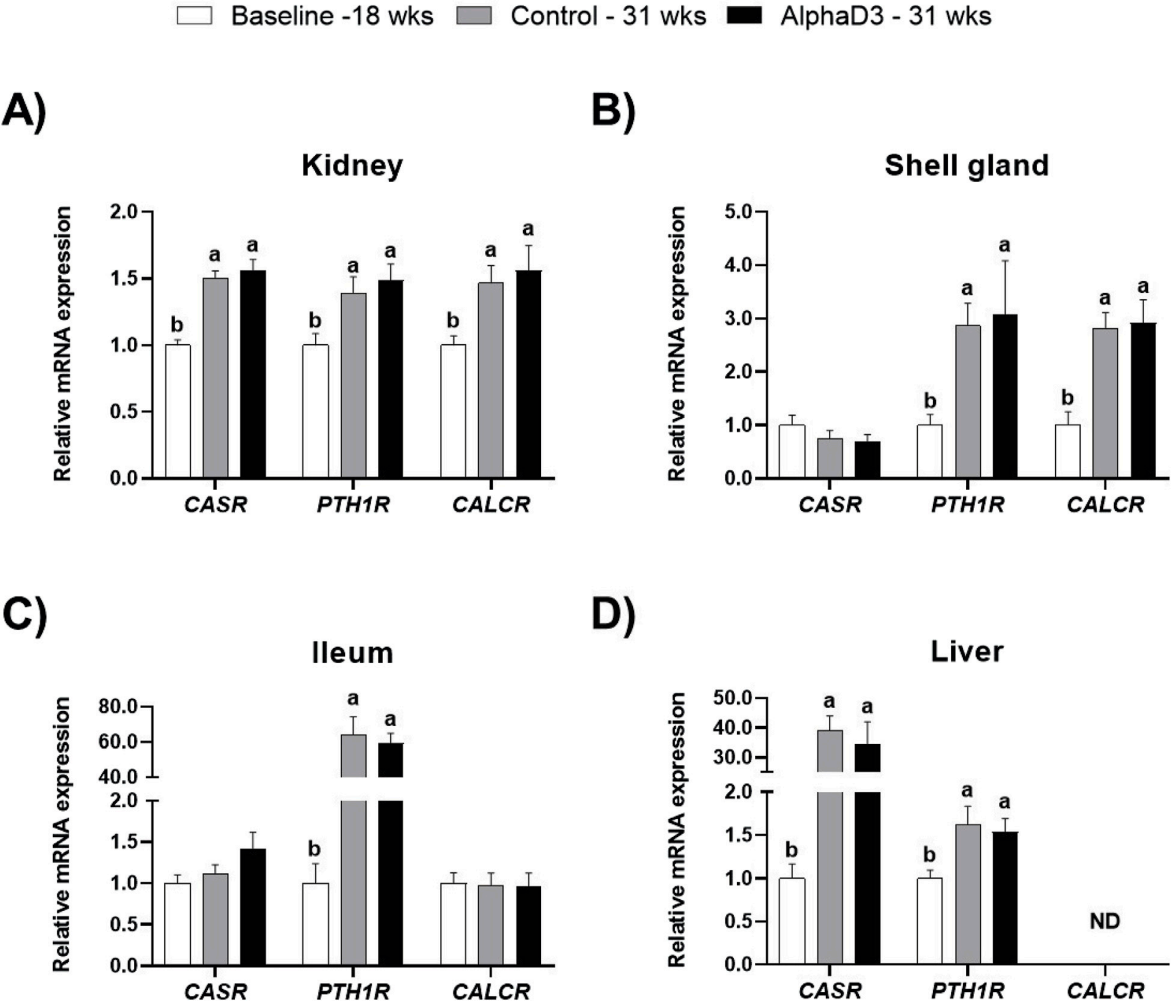
Expression profiles of receptors mediating hormonal regulation of calcium homeostasis. Levels of mRNA for *CASR*, *PTH1R*, and *CALCR* were determined in **(A)** kidney, **(B)** shell gland, **(C)** ileum, and **(D)** liver at 18 weeks (Baseline) in hens fed a single developer diet and 31 weeks in hens fed either a control or AlphaD3 (1α-hydroxycholecalciferol)- supplemented diet. Relative expression of mRNA was normalized to *GAPDH* mRNA in kidney and ileum, *18S* rRNA in shell gland, and *CYCLO* mRNA in liver. Values (mean +SEM) are expressed relative to the 18 weeks baseline group (equivalent to 1). Within each gene, different letters indicate values are significantly different between groups (*p* ≤ 0.05; n = 8 hens/group). ND, not detected.

To evaluate changes in hormonal signaling associated with calcium homeostasis after the onset of lay, mRNA expression levels of *CASR, PTH1R,* and *CALCR* were measured in kidney, shell gland, ileum, and liver. Though no significant differences between diets were found at 31 weeks, there were several changes that occurred between 18 and 31 weeks in these tissues. Expression of all three genes was upregulated in kidney at 31 weeks (*p* ≤ 0.05; Figure 1A). In shell gland, *CASR* expression did not change with age, but both *PTH1R* and *CALCR* were higher at 31 weeks (*p* ≤ 0.05; Figure 1B). While expression of both *CASR* and *CALCR* did not differ between 18 and 31 weeks in ileum (*p* > 0.05), significant increases of approximately 60-fold were observed for *PTH1R* at 31 weeks in this tissue (*p* ≤ 0.05; Figure 1C). In liver, levels of *CASR* and *PTH1R* were found to be increased at 31 weeks, with *CASR* exhibiting an almost 40-fold increase (*p* ≤ 0.05; Figure 1D); however, *CALCR* was not detected in this tissue (Figure 1D).

#### 3.1.2 Phosphorus homeostasis

**FIGURE 2:**
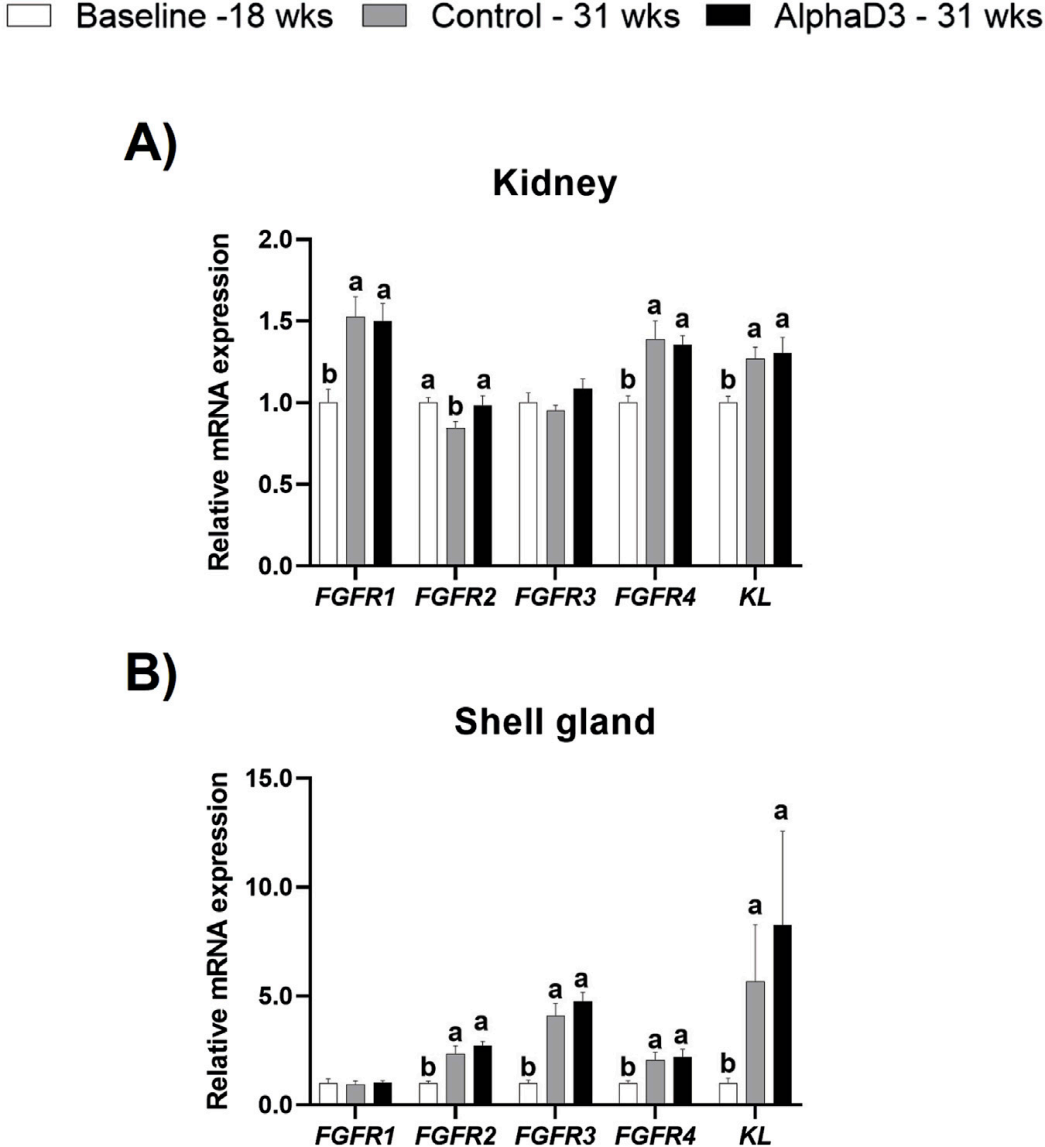
Expression profile of receptors mediating hormonal regulation of phosphorus homeostasis. Levels of mRNA for *FGFR1, FGFR2, FGFR3, FGFR4*, and *KL* were determined in **(A)** kidney and **(B)** shell gland at 18 weeks (Baseline) in hens fed a single developer diet and 31 weeks in hens fed either a control or AlphaD3 (1α-hydroxycholecalciferol)- supplemented diet. Relative expression of mRNA was normalized to *GAPDH* mRNA in kidney and *18S* rRNA in shell gland. Values (mean +SEM) are expressed relative to the baseline group (equivalent to 1). Within each gene, different letters indicate values are significantly different between groups (*p* ≤ 0.05; n = 8 hens/group).

To identify changes in FGF23 hormonal sensitivity after the onset of lay, expression of *FGFR1, FGFR2, FGFR3*, *FGFR4,* and the co-receptor *KL* were measured in kidney, a known target tissue for FGF23 signaling, and shell gland based on our previous findings associated with phosphorus utilization and signaling in this tissue (Sinclair-Black et al., 2024). Renal expression of *FGFR1, FGFR4*, and *KL* was found to be upregulated at 31 weeks (*p* ≤ 0.05) but did not differ between diets (*p* > 0.05; Figure 2A). Interestingly, *FGFR2* was found to be significantly downregulated at 31 weeks only in the control hens, with AlphaD3 hens maintaining expression. This resulted in significant differences between hens fed different diets at 31 weeks (*p* ≤ 0.05; Figure 2A). Renal expression of *FGFR3* did not exhibit changes after the onset of lay, nor was it influenced by diet (*p* > 0.05; Figure 2A). Though differences were not evident between control- and AlphaD3-fed hens at 31 weeks (*p* > 0.05), levels of *FGFR2, FGFR3, FGFR4,* and *KL* were found to be upregulated at 31 weeks in the shell gland, with *KL* exhibiting an increase of approximately 7-fold (*p* ≤ 0.05; Figure 2B). However, no age- or diet-induced changes were detected for *FGFR1* in this tissue (*p* > 0.05; Figure 2B). These results indicate that hens acquire greater sensitivity to hormones responsible for regulating calcium and phosphorus homeostasis after the onset of lay, potentially as a mechanism to facilitate eggshell mineralization and medullary bone formation. Furthermore, upregulation of the expression of FGF23 receptors after the onset of lay in the shell gland suggests this tissue may be a novel target for FGF23 signaling.

### 3.2 Vitamin D_3_ metabolism and action

#### 3.2.1 Vitamin D_3_ hydroxylation

**FIGURE 3:**
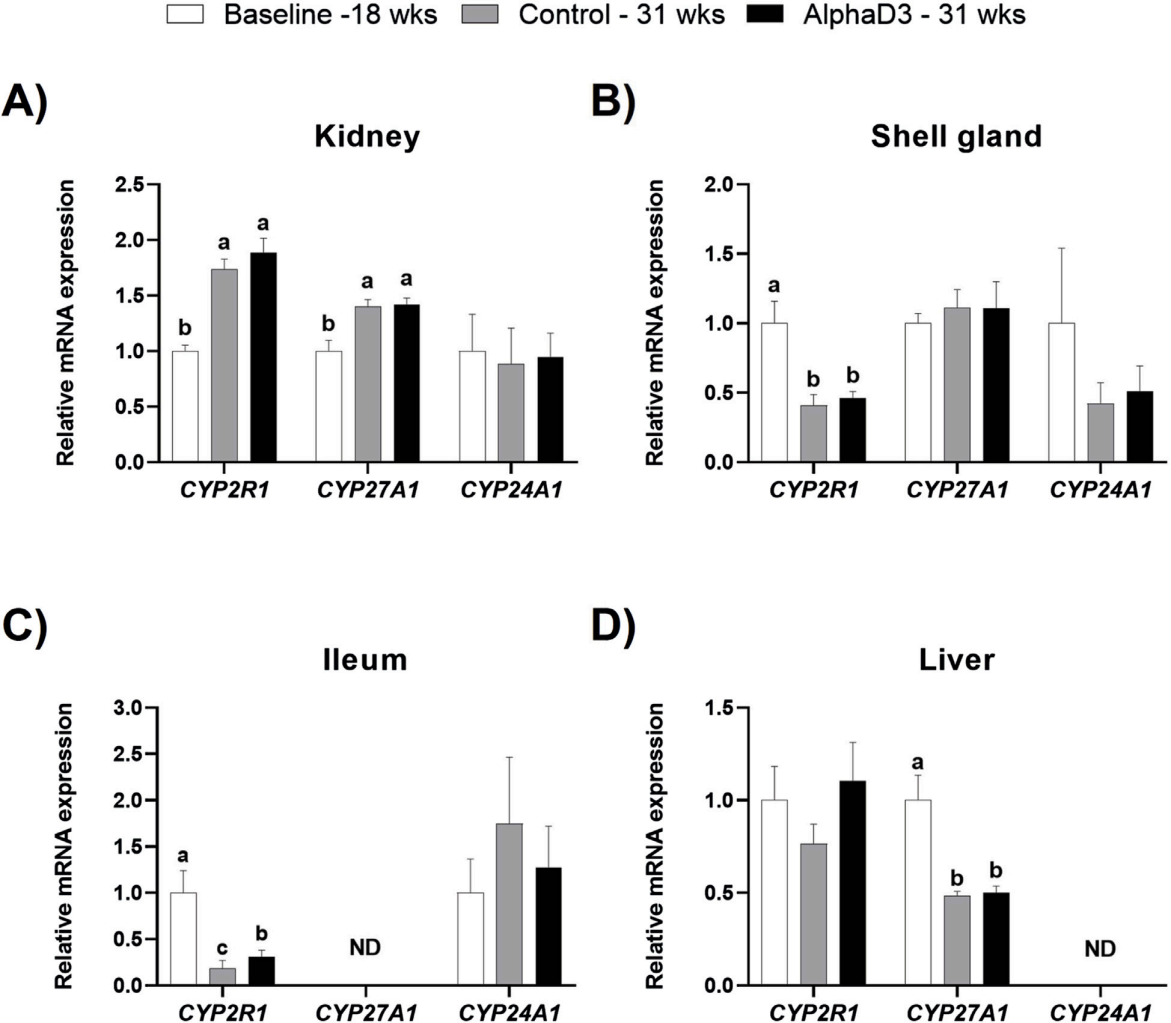
Expression profiles of enzymes regulating vitamin D_3_ metabolism. Levels of mRNA for *CYP2R1, CYP27A1,* and *CYP24A1* were determined in **(A)** kidney, **(B)** shell gland, **(C)** ileum, and **(D)** liver at 18 weeks (Baseline) in hens fed a single developer diet and 31 weeks in hens fed either a control or AlphaD3 (1α-hydroxycholecalciferol)- supplemented diet. Relative expression of mRNA was normalized to *GAPDH* mRNA in kidney and ileum, *18S* rRNA in shell gland, and *CYCLO* mRNA in liver. Values (mean +SEM) are expressed relative to the baseline group (equivalent to 1). Within each gene, different letters indicate values are significantly different between groups (*p* ≤ 0.05; n = 8 hens/group). ND, not detected.

To determine how the onset of lay may influence the synthesis of vitamin D_3_ metabolites, expression of enzymes responsible for the conversion of cholecalciferol into its active or inactive forms was analyzed in kidney, shell gland, ileum, and liver. Expression of two 25-hydroxylase enzymes encoded by *CYP2R1* or *CYP27A1* were upregulated in kidney at 31 weeks compared to 18 weeks (*p* ≤ 0.05), with no significant differences between diets at 31 weeks (*p* > 0.05; Figure 3A). The opposite age effect was observed for *CYP2R1* levels in the shell gland, where expression was downregulated after the onset of lay (*p* ≤ 0.05), although no differences between diets were observed at 31 weeks (*p* > 0.05; Figure 3B). There were no significant changes observed for *CYP27A1* in shell gland (*p* > 0.05; Figure 3B). Although ileal expression of *CYP2R1* decreased after the onset of lay in both groups, AlphaD3-supplemented hens exhibited higher expression levels when compared to control hens at 31 weeks (*p* ≤ 0.05; Figure 3C). Unlike the other tissues, hepatic *CYP2R1* was not significantly influenced by age or diet (*p* > 0.05), while levels of *CYP27A1* in liver were downregulated after the onset of lay (*p* ≤ 0.05), with no differences between diets observed at 31 weeks (*p* > 0.05; Figure 3D). Inactivation of vitamin D_3_ is mediated by the 24-hydroxylase enzyme encoded by *CYP24A1*, and no significant effects of age or diet were observed for any of the tissues where it was detected (*p* > 0.05; Figures 3A–D). Expression of *CYP27A1* and *CYP24A1* was not detected in ileum and liver, respectively. These results suggest that changes in expression of 25-hydroxylase genes occur in several tissues after the onset of lay and indicate that, in addition to liver, kidney, shell gland, and ileum might also play a role in the 25-hydroxylation of dietary vitamin D_3_.

#### 3.2.2 Circulating vitamin D_3_ metabolites

**FIGURE 4:**
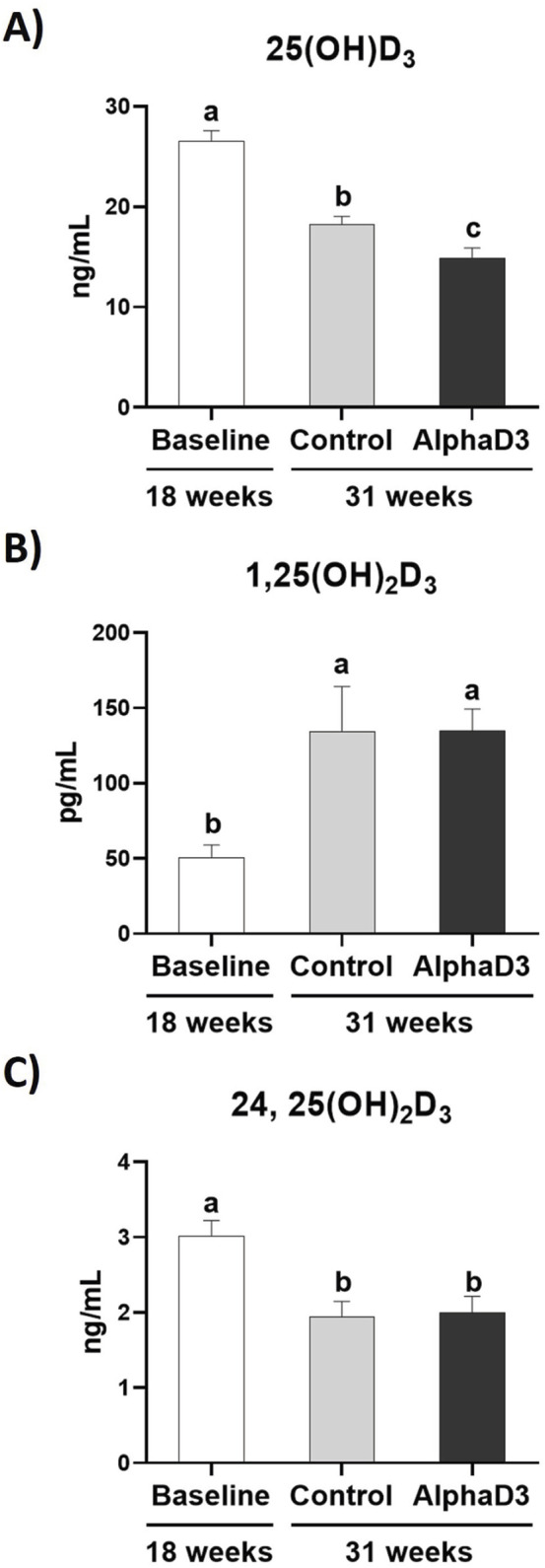
Circulating vitamin D3 metabolites. Levels of the circulating vitamin D3 metabolites **(A)** 25(OH)D_3_ (ng/mL), **(B)** 1,25(OH)_2_D_3_, and **(C)** 24,25(OH)_2_D_3_ were measured at 18 weeks (Baseline) in hens fed a single developer diet and 31 weeks in hens fed either a control or AlphaD3 (1α-hydroxycholecalciferol)- supplemented diet. Means (+SEM) with different letters are significantly different between groups (*p* ≤ 0.05; n = 8 hens/group).

In addition to measuring mRNA expression, circulating levels of each vitamin D_3_ metabolite were measured in plasma. Circulating 25(OH)D_3_ significantly decreased in both groups at 31 weeks when compared to 18 weeks (*p* ≤ 0.05), with AlphaD3-supplemented hens exhibiting significantly lower plasma levels when compared to control hens (*p* ≤ 0.05; Figure 4A). In contrast, plasma levels of 1,25(OH)_2_D_3_ were higher at 31 weeks (*p* ≤ 0.05), though no difference between diets was found (*p* ≤ 0.05; Figure 4B). Circulating levels of 24,25(OH)_2_D_3_ declined at 31 weeks (*p* ≤ 0.05), again with no differences observed between diets at 31 weeks (*p* ≤ 0.05; Figure 4C). Respective changes in these vitamin D_3_ metabolites after the onset of lay indicate that higher levels of active 1,25(OH)_2_D_3_ and lower levels of inactive 24,25(OH)_2_D_3_ are likely important for the increased capacity to utilize calcium and phosphorus for egg production.

#### 3.2.3 Vitamin D_3_ signaling

**FIGURE 5:**
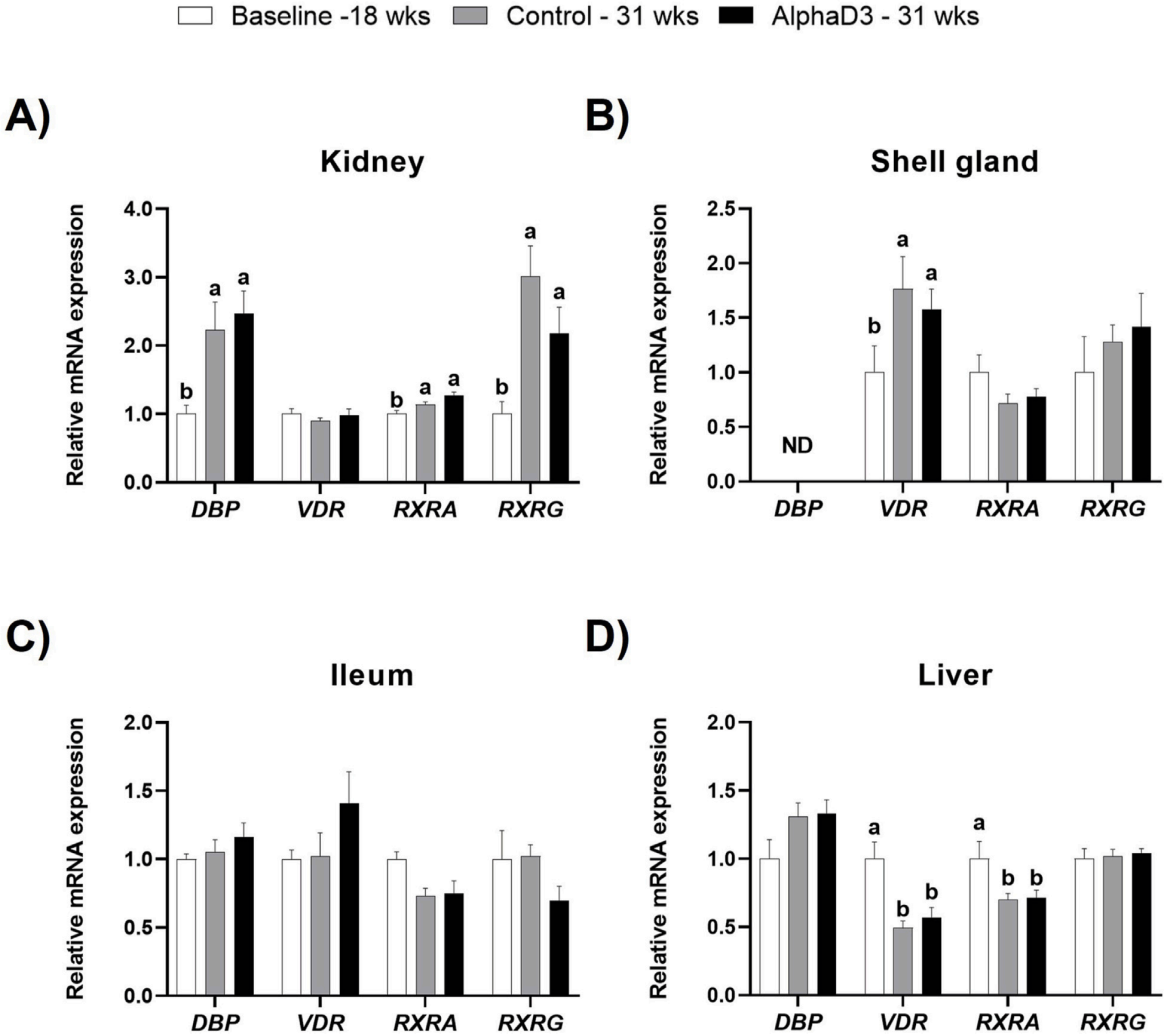
Expression profiles of vitamin D_3_ genomic actions. Levels of mRNA for *DBP*, *VDR, RXRA,* and *RXRG* were determined in **(A)** kidney, **(B)** shell gland, **(C)** ileum, and **(D)** liver at 18 weeks (Baseline) in hens fed a single developer diet and 31 weeks in hens fed either a control or AlphaD3 (1α-hydroxycholecalciferol)- supplemented diet. Relative expression of mRNA was normalized to *GAPDH* mRNA in kidney and ileum, *18S* rRNA in shell gland, and *CYCLO* mRNA in liver. Values (mean +SEM) are expressed relative to the baseline group (equivalent to 1). Within each gene, different letters indicate values are significantly different between groups (*p* ≤ 0.05; n = 8 hens/group). ND, not detected.

In circulation, all three vitamin D_3_ metabolites are bound by vitamin D binding protein (**DBP**), and genomic signaling initiated by 1,25(OH)_2_D_3_ that regulates calcium and phosphorus homeostasis is mediated by vitamin D receptor (***VDR***) and its heterodimeric transcriptional partners, retinoid-X-receptor alpha (***RXRA***) or gamma (***RXRG***). Hence, mRNA levels of *DBP*, *VDR, RXR,* and *RXRG* were measured in kidney, shell gland, ileum, and liver to evaluate how vitamin D_3_ transport and signaling change after the onset of lay. At 31 weeks, significant differences between diets were not detected for these genes in any tissue (*p* > 0.05); however, differences before and after the onset of lay were apparent (Figure 5). Although renal levels of *VDR* were not significantly affected by age (*p* > 0.05), expression of *DBP, RXRA*, and *RXRG* was found to be upregulated at 31 weeks (*p* ≤ 0.05; Figure 5A). Unlike other tissues, *DBP* expression was not detected in the shell gland, and while levels of *VDR* were found to increase after the onset of lay (*p* ≤ 0.05), no influence of age was observed for either *RXRA* or *RXRG* (*p* > 0.05; Figure 5B). In ileum, no significant differences were found for any of the genes (*p* > 0.05; Figure 5C). Furthermore, although no changes were observed for hepatic *DBP* and *RXRG* (*p* > 0.05), both *VDR* and *RXRA* were found to be downregulated in liver after the onset of lay (*p* ≤ 0.05; Figure 5D). Taken together, it appears that kidney and shell gland become more responsive to 1,25(OH)_2_D_3_ signaling after the onset of lay, while ileum seems to have a similar sensitivity before and after egg production begins. Furthermore, although DBP is mainly synthesized in the liver, upregulation of renal *DBP* after the onset of lay could facilitate an increase in local concentration of vitamin D_3_ metabolites and 1,25(OH)_2_D_3_ signaling.

### 3.3 Mineral uptake and utilization

#### 3.3.1 Calcium and bicarbonate transport

**FIGURE 6:**
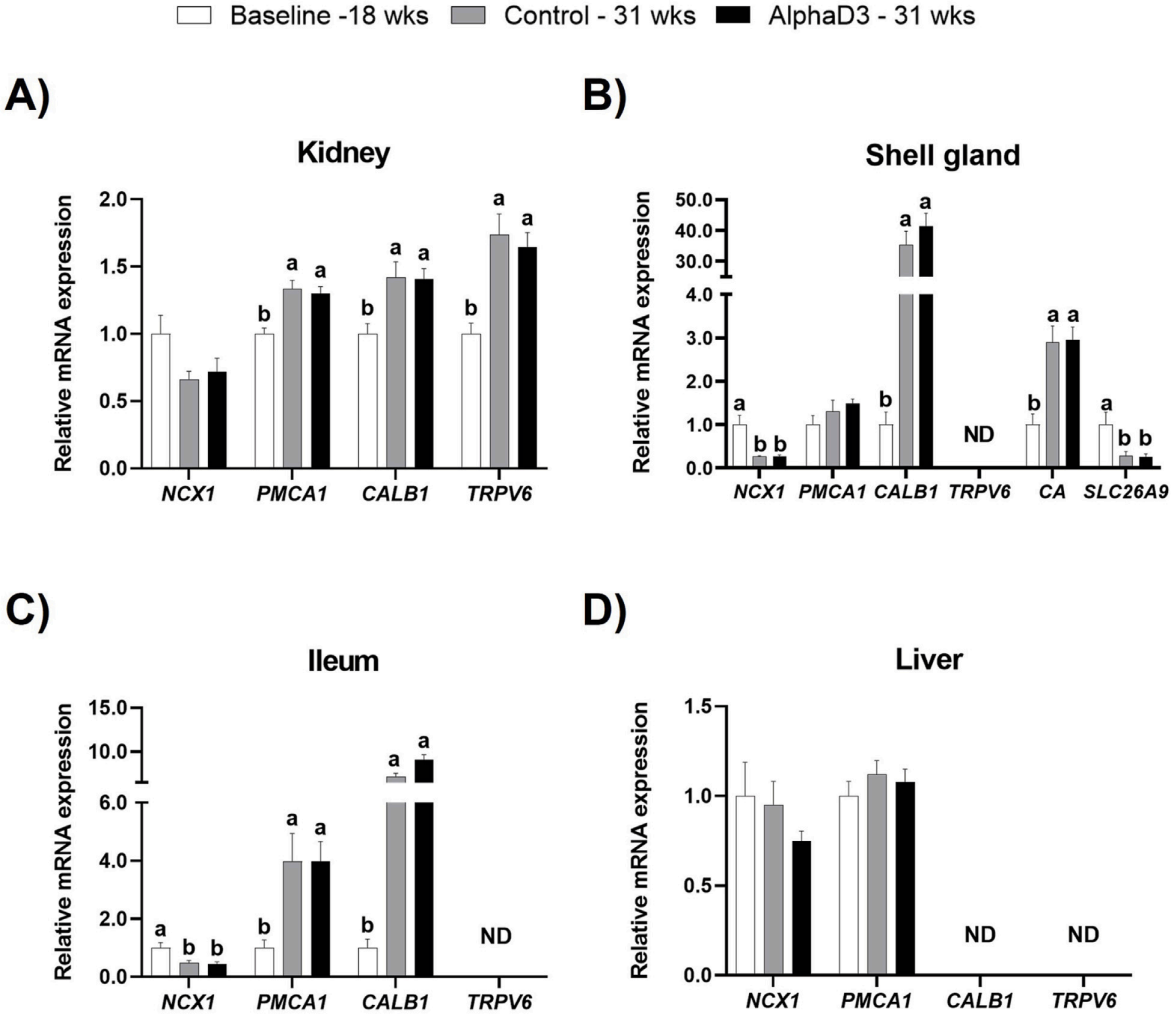
Expression profiles of calcium and bicarbonate uptake and utilization. Expression levels of mRNA for *NCX1, PMCA1, CALB1*, and *TRPV6* were determined in **(A)** kidney, **(B)** shell gland, **(C)** ileum, **(D)** liver, and *SLC26A9* and *CA2* in **(B)** shell gland at 18 weeks (Baseline) in hens fed a single developer diet and 31 weeks in hens fed either a control or AlphaD3 (1α-hydroxycholecalciferol)- supplemented diet. Relative expression of mRNA was normalized to *GAPDH* mRNA in kidney and ileum, *18S* rRNA in shell gland, and *CYCLO* mRNA in liver. Values (mean +SEM; n = 8 hens/group) are expressed relative to the baseline group (equivalent to 1). Within each gene, different letters indicate values are significantly different between groups (*p* ≤ 0.05; n = 8 hens/group). ND, not detected.

In order to determine what genes might be involved in mediating calcium uptake and utilization associated with the onset of lay, plasma membrane calcium transporters sodium-calcium exchanger 1 (***NCX1***), ATPase plasma membrane calcium transporting 1 *(* ***PMCA1***), and transient receptor potential cation channel subfamily V member 6 *(* ***TRPV6***), as well as the intracellular calcium chaperone calbindin (***CALB1***), were measured in kidney, shell gland, ileum, and liver. Several transport proteins were found to be upregulated after the onset of lay; however, no differences were detected between diets at 31 weeks (*p* > 0.05; Figure 6). Although renal *NCX1* was not impacted by age (*p* > 0.05), expression of *PMCA1*, *TRPV6*, and *CALB1* increased at 31 weeks (*p* ≤ 0.05; Figure 6A). In shell gland, *CALB1* was found to have the highest upregulation after the onset lay with a 40-fold increase (*p* ≤ 0.05). Interestingly, *NCX1* was found to decrease after the onset of lay (*p* ≤ 0.05), and *PMCA1* expression was not influenced by age (*p* > 0.05; Figure 6B). Similar to shell gland, ileal expression of *NCX1* was downregulated at 31 weeks; however, *PMCA1* and *CALB1* levels were found to increase, with *CALB1* exhibiting a 10-fold increase (*p* ≤ 0.05; Figure 6C). No significant changes were observed in liver for *NCX1* or *PMCA1* (*p* > 0.05; Figure 6D). Expression of *TRPV6* was not detected in shell gland or ileum, and neither *TRPV6* or *CALB1* were detected in liver.

The structure of the eggshell is predominantly composed of calcium carbonate crystals, which requires the action of carbonic anhydrase (**CA2**), as well as the presence of bicarbonate ions, which are mobilized to the shell gland lumen by the solute carrier family 26 member 9 (***SLC26A9***). Hence, to evaluate the changes associated with eggshell formation in the shell gland after the onset of lay, mRNA expression levels of *CA2* and *SLC26A9* were evaluated. No differences were observed between diets at 31 weeks for either of the genes evaluated (*p* > 0.05); however, *CA2* exhibited an increase after the onset of lay, while *SLC26A9* was found to be downregulated at 31 weeks (*p* ≤ 0.05; Figure 6B). These results indicate an acquired capacity to transfer calcium in kidney, shell gland, and intestine, as well as the ability to synthesize bicarbonate necessary for eggshell formation after the onset of lay.

#### 3.3.2 Phosphorus transport

**FIGURE 7:**
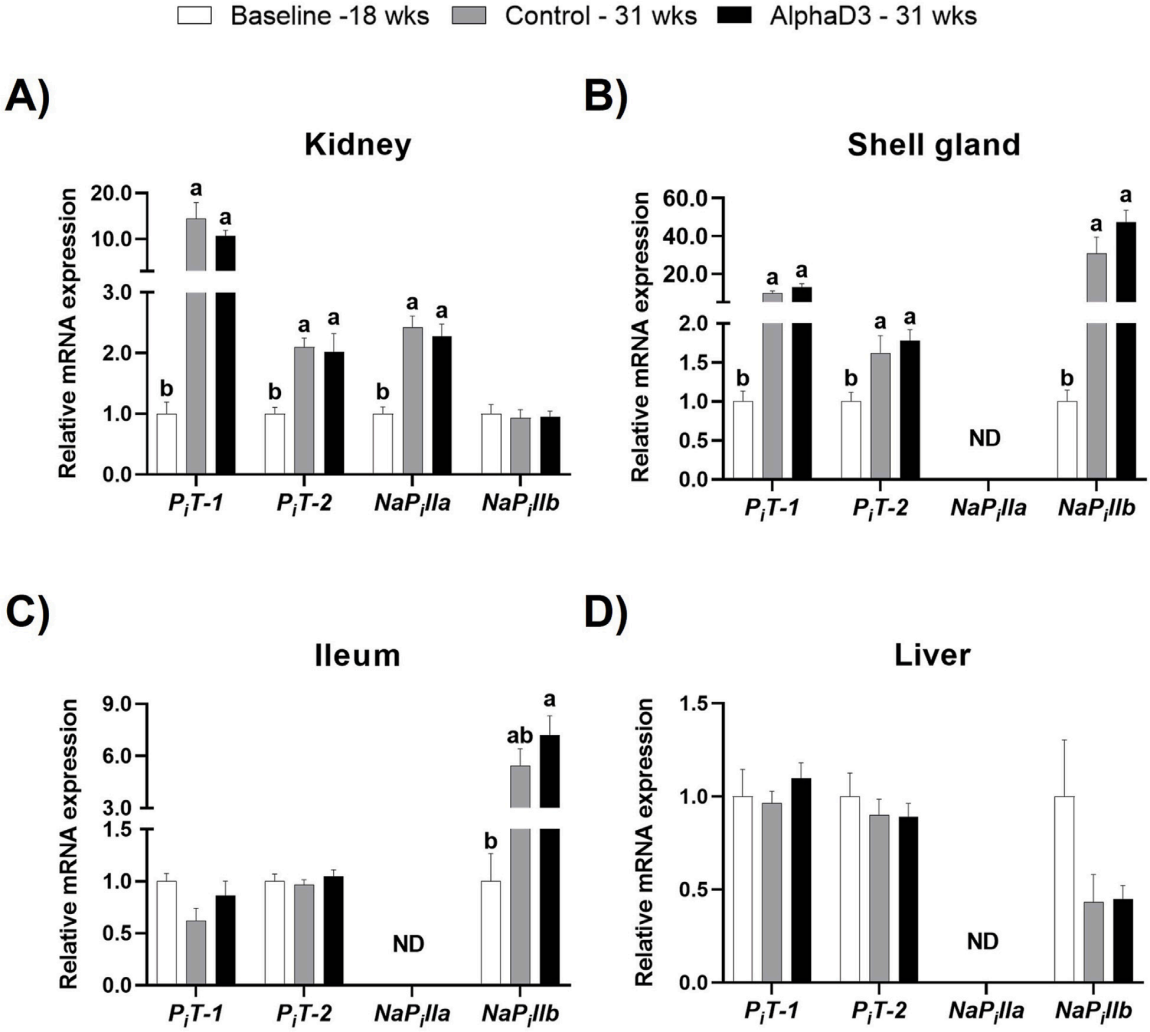
Expression profiles of phosphorus uptake and utilization. Expression levels of mRNA for *P*_*i*_ *T-1, P*_*i*_ *T-2, NaP*_*i*_ *lla,* and *NaP*_*i*_ *llb* were determined in **(A)** kidney, **(B)** shell gland, **(C)** ileum, and **(D)** liver at 18 weeks (Baseline) in hens fed a single developer diet and 31 weeks in hens fed either a control or AlphaD3 (1α-hydroxycholecalciferol)- supplemented diet. Relative expression of mRNA was normalized to *GAPDH* mRNA in kidney and ileum, *18S* rRNA in shell gland, and *CYCLO* mRNA in liver. Values (mean +SEM) are expressed relative to the baseline group (equivalent to 1). Within each gene, different letters indicate values are significantly different between groups (*p* ≤ 0.05; n = 8 hens/group). ND, not detected.

To evaluate changes in phosphorus uptake and utilization after the onset of lay, plasma membrane phosphorus transporters inorganic phosphorus transporter 1 (***P***_***i***_ ***T-1***) and 2 (***P***_***i***_ ***T-2***) and sodium-dependent phosphate transporter IIa (***NaP***_***i***_ ***lla***) and IIb (***NaP***_***i***_ ***llb***) were measured in kidney, shell gland, ileum, and liver. No differences were observed between diets at 31 weeks. Renal levels of *P*_*i*_ *T-1, P*_*i*_ *T-2*, and *NaP*_*i*_ *lla* were upregulated in kidney at 31 weeks, with *P*_*i*_ *T-1* showing the largest increase with a 10-fold change (*p* ≤ 0.05; Figure 7A). The phosphorus transporter *NaP*_*i*_ *llb*, which was only detected in kidney, did not exhibit any changes (*p* > 0.05; Figure 7A). In the shell gland, expression of *P*_*i*_ *T-1, P*_*i*_ *T-2*, and *NaP*_*i*_ *llb* was higher at 31 weeks, with *P*_*i*_ *T-1* and *NaP*_*i*_ *llb* exhibiting increases of 12- and 40-fold, respectively (*p* ≤ 0.05; Figure 7B). Ileal *NaP*_*i*_ *llb* was the only phosphorus transporter with a significant increase of approximately 7-fold at 31 weeks (*p* ≤ 0.05; Figure 7C). Liver did not exhibit any significant changes in these transporters after the onset of lay (*p* > 0.05; Figure 7D). Upregulation of select phosphorus transporters after the onset of lay likely indicates greater capacity of several tissues to transfer and utilize phosphorus. Specifically, *P*_*i*_ *T-1* appears to contribute largely to the renal excretion of phosphorus necessary after bone resorption to facilitate eggshell formation. In the shell gland, both *P*_*i*_ *T-1* and *NaP*_*i*_ *llb* likely play a major role in phosphorus utilization during late eggshell formation, while *NaP*_*i*_ *llb* seems to assist intestinal uptake in the ileum.

### 3.4 Changes in skeletal parameters after the onset of lay

**FIGURE 8:**
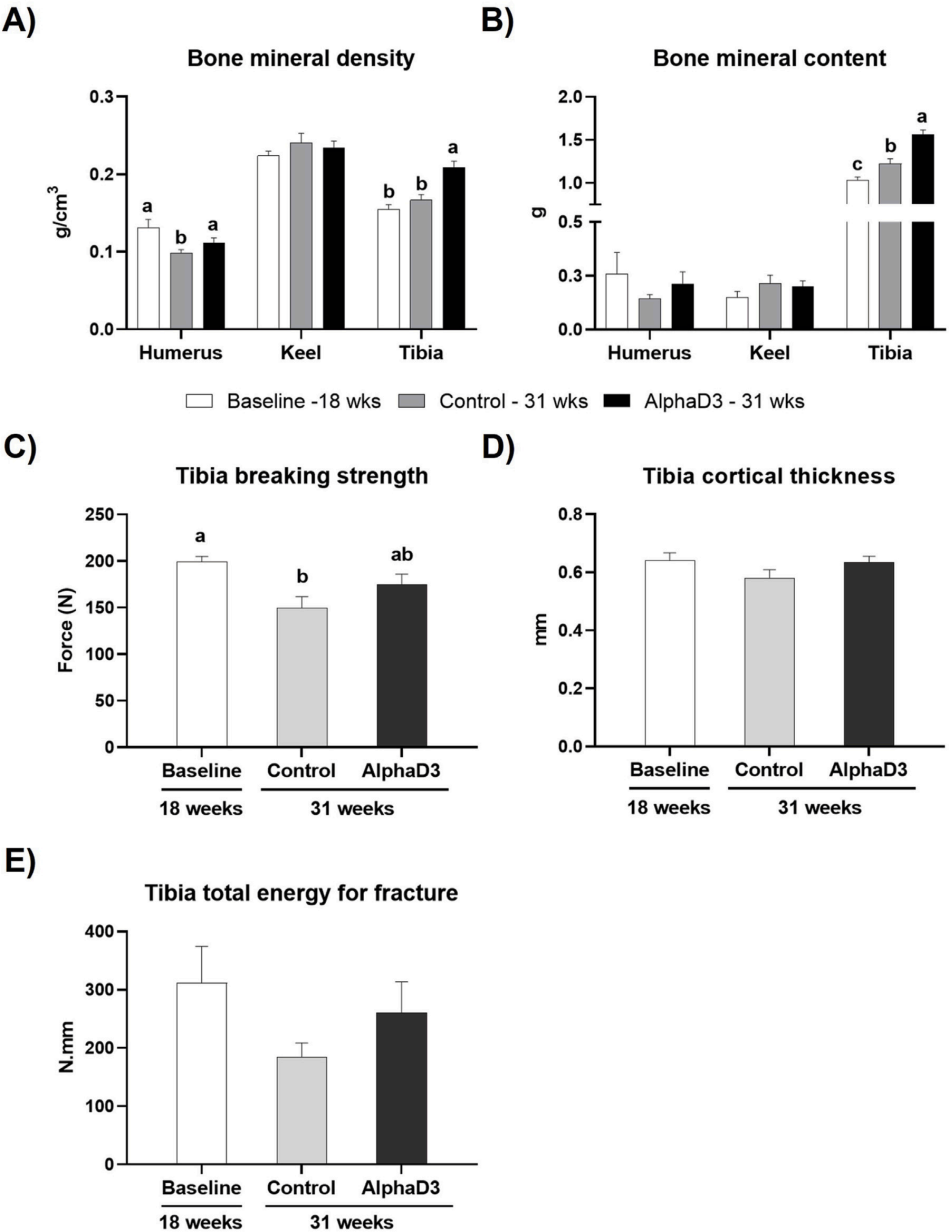
Changes in skeletal parameters after the onset of lay. **(A)** Bone mineral density (g/cm^3^) and **(B)** bone mineral content (g) was determined for humerus, keel bone, and tibia, and **(C)** breaking strength (N), **(D)** cortical thickness (mm), and **(E)** Tibia total energy for fracture (N.mm) were determined in tibia. Each parameter was measured at 18 weeks (Baseline) in hens fed a single developer diet and 31 weeks in hens fed either a control or AlphaD3 (1α-hydroxycholecalciferol)- supplemented diet. Within each bone **(A, B)** or graph **(C, D, E)**, values (mean +SEM) with different letters are significantly different between groups (*p* ≤ 0.05; n = 8 hens/group).

To evaluate changes in bone mineralization and integrity in relation to medullary bone formation and the onset of lay, BMD and BMC were measured in humerus, keel bone, and tibia and breaking strength, cortical thickness, and AUC were determined for tibia. Keel bone BMD and BMC were not significantly impacted by age or diet (*p* > 0.05; Figures 8A,B). While humeral BMD was found to be lower in the control-fed hens at 31 weeks when compared to the other two groups, tibial BMD was higher for AlphaD3-supplemented hens when compared to both control-fed hens at 31 weeks and hens at 18 weeks (*p* ≤ 0.05; Figure 8A). Similarly, although no changes were observed in humerus BMC (*p* > 0.05), onset of lay increased tibial BMC so that levels in both groups of 31-week-old hens were significantly higher than those at 18 weeks (*p* ≤ 0.05), with hens fed the AlphaD3-supplemented diet having higher tibial BMC than control-fed hens (*p* ≤ 0.05; Figure 8B). Furthermore, tibial breaking strength was found to decrease after the onset of lay for the control group, while AlphaD3-supplemented hens were able to maintain breaking strength at intermediate levels (*p* ≤ 0.05; Figure 8C). No differences were found for tibial cortical thickness and AUC (*p* > 0.05; Figures 8D,E). Taken together, these results indicate that supplementation with AlphaD3 may improve bone mineral deposition during the critical period of medullary bone formation and help maintain bone strength after the onset of lay.

In order to determine changes in keel bone parameters associated with the onset of lay, variables indicatives of keel bone deformation and damage were scored. Prevalence and severity of keel bone deviation and fracture prevalence were measured. There were no statistically significant differences observed for any of the parameters evaluated (*p* > 0.05; Table 4). Interestingly, even hens prior to the onset of lay exhibited a deviation prevalence of 62.5%, and this increased at 31 weeks to 75% and 87.5% in control and AlphaD3-supplemented hens, respectively.

**TABLE 4:** Keel bone deviation and fracture score distributions across experimental groups.

	Baseline		Control		AlphaD3		
	18 weeks		31 weeks		31 weeks		
	n	%	n	%	n	%	*p*-value
Deviation prevalence	5/8	62.5 ± 6.05	6/8	75.0 ± 5.41	7/8	87.5 ± 4.13	0.5279
Deviation severity							0.4079
0 (Straight)	3/8	37.5 ± 6.05	2/8	25.0 ± 5.41	1/8	12.5 ± 4.13	
1 (Mild)	5/8	62.5 + 6.05	5/8	62.5 ± 6.05	6/8	75.0 ± 5.41	
2 (Severe)	0/8	0.0 ± 0.0	1/8	12.5 ± 4.13	1/8	12.5 ± 4.13	
Fracture prevalence	0/8	0.0 ± 0.0	1/8	12.5 ± 4.13	0/8	0.0 ± 0.0	0.3679

## 4 Discussion

**FIGURE 9:**
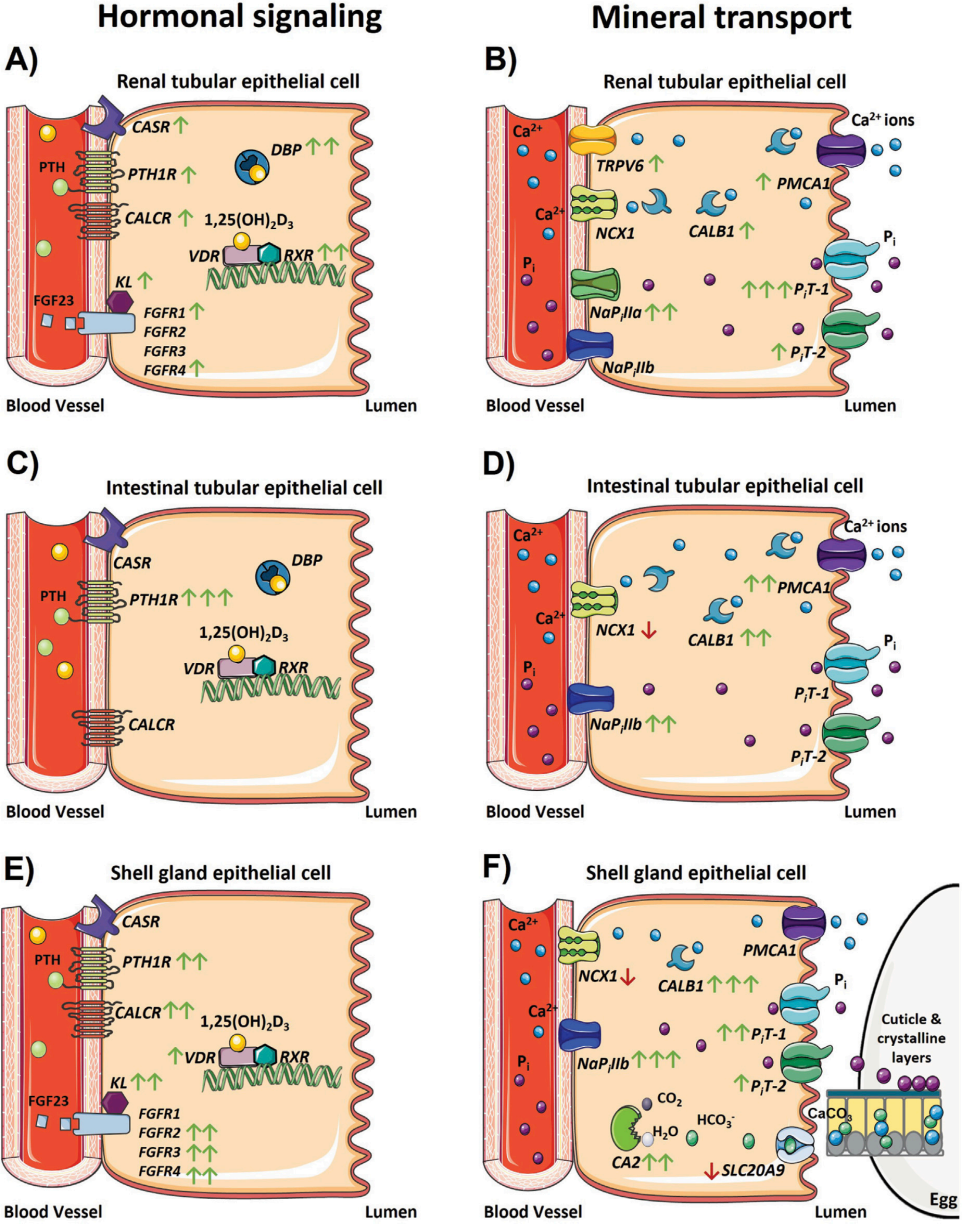
Changes in the physiological regulation of calcium and phosphorus homeostasis after the onset of egg production. Models depicting changes in gene expression associated with hormonal signaling regulating calcium and phosphorus homeostasis and transport that occur in the **(A, B)** kidney, **(C, D)** ileum, and **(E, F)** shell gland during the onset of lay. Green and red arrows indicate genes that were significantly increased or decreased in expression between 18 and 31 weeks, respectively, and the number of arrows represents the magnitude of the changes. One arrow indicates a fold change between 1 and 2; two arrows indicate a fold change between 2 and 10; and three arrows indicate a fold change greater than 10. The absence of any arrow indicates no significant changes were detected between 18 and 31 weeks. The absence of a gene depicted in each model indicates its expression was not detected in that tissue, with the exception of *FGFR1 – 4* and *KL* in the ileum because these genes were not analyzed in that tissue. Parts of the figure were created using pictures from Servier Medical Art (https://smart.servier.com/), licensed under a Creative Commons Attribution 4.0 Unported License (https://creativecommons.org/licenses/by/4.0/).

At the onset of egg production, laying hens undergo many physiological changes necessary to facilitate uptake, storage, and utilization of large amounts of calcium for eggshell calcification. These start to occur around 2 weeks prior to the onset of lay and involve specialized tissues such as intestine, kidney, and shell gland that play major roles in calcium and phosphorus absorption, retention, and utilization (Bar, 2008). This study identified potential physiological mechanisms related to hormonal signaling and mineral transport in these tissues by measuring gene expression, as well as evaluated bone parameters prior to and after the onset of lay. The effect of dietary supplementation with AlphaD3 on these changes was also evaluated. Changes in gene expression were evaluated by measuring mRNA levels without further examination into whether protein levels of the same transcripts changed in a similar manner. Since variations in mRNA expression do not necessarily reflect functional modifications at the cellular level, caution should be exercised when interpreting the results. However, these data are useful at identifying systems and networks potentially involved in mediating changes that allow for enhanced handling of minerals necessary to support daily egg production. Transcriptional findings suggest PTH could be a major hormonal driver regulating calcium homeostasis across all tissues following the onset of lay, and this is particularly apparent in ileum and shell gland. Similarly, hormonal sensitivity to FGF23 appears to be upregulated after the onset of lay in kidney and shell gland, with the latter being identified as a novel target of this hormone. In addition to its role in mineral retention and excretion, kidney could be involved in the 25-hydroxylation of vitamin D_3_ and DBP synthesis. Calbindin was demonstrated to have a primary role in assisting with calcium flux in all tissues, especially the shell gland, while calcium transporter *NCX1* does not seem to be influenced by these adaptative changes in any of the tissues examined. Phosphorus excretion and absorption appears to occur primarily through *P*_*i*_ *T-1* in kidney and *NaP*_*i*_ *llb* in the ileum, respectively. Similarly, phosphorus utilization for hydroxyapatite and cuticle deposition within the eggshell seems to be mediated by *P*_*i*_ *T-1* and *NaP*_*i*_ *llb* in the shell gland. The above findings related to hormonal signaling and mineral transport are depicted in Figure 9. It should be noted that hens were only sampled prior to lay at 18 weeks and during peak production at 31 weeks. Including ages between these would allow for a more comprehensive examination of the transition that occurs immediately around the onset of lay. Most mRNA expression profiles appeared unaffected by dietary supplementation with AlphaD3, a finding that could be result of the high efficiency with which hens at peak production are able handle the daily mineral turnover necessary for eggshell mineralization. Including intermediate ages during early lay may have revealed ways in which tissues other than bone were influenced by AlphaD3 in the diet. Despite few observable impacts on mRNA levels in the tissues examined, several bone mineralization parameters were improved by feeding hens AlphaD3 during the critical period of medullary bone formation between 18 and 31 weeks of age.

Hormonal regulation of calcium homeostasis is primarily mediated by the synchronized activity of CASR, PTH, and CALC, though specific involvement of each of these during the onset of lay is not fully understood. Observations from this study found expression of *CASR* to change between 18- and 31-week in kidney and liver, with liver exhibiting a pronounced 60-fold increase. Although renal involvement in calcium homeostasis has been widely described (Sommerville and Fox, 1987; Wideman, 1987), liver is not canonically associated in this process beyond 25-hydroxylation of vitamin D_3_ (Shang et al., 2023). CASR is not specific to calcium and has been demonstrated to contain binding sites for other divalent ions including magnesium (Quinn et al., 2013), zinc, and iron (Chanpaisaeng et al., 2021), as well as phosphate (Centeno et al., 2019) and aromatic amino acids (Conigrave et al., 2000). Hence, it is possible that hepatic expression of this gene may be related to the vitellogenesis and influenced by estrogen levels (Gloux et al., 2019; Cui et al., 2020). There are very few studies in chickens related to hepatic expression of CASR and, in contrast to this study, Deng et al. (2010) reported that this gene was not detected in liver or other tissues such as heart, spleen, lungs, pancreas, or stomach of laying hens. However, hepatic expression of this gene has been shown in mammals, and studies with mouse hepatocytes demonstrated CASR’s capability to sense and mobilize calcium associated with bile secretion (Canaff et al., 2001) and induce intracellular calcium elevation (Xing et al., 2010).

The influence of PTH on regulation of calcium and phosphorus uptake and utilization was demonstrated in this study, where all tissues evaluated had heightened *PTH1R* mRNA expression after the onset of lay, an event that was only observed for this gene. Although function of PTH have been well described in kidney (Wideman, 1987; Elaroussi et al., 1993), the most apparent upregulation in this study was observed in ileum. For decades, the influence of PTH on intestinal calcium and phosphorus absorption has been described as indirect, as it occurs primarily via activation of 1,25(OH)_2_D_3_ (Tanaka et al., 1971; Bar et al., 1990). However, other authors have suggested that PTH may directly stimulate calcium absorption in mice (Picotto et al., 1997), fish (Rotllant et al., 2006), and chickens (Nemere and Norman, 1986). Thus, ileal upregulation of *PTH1R* in this study could indicate a direct effect of PTH on the intestine to meet increasing calcium demands after the onset of lay.

In the case of shell gland, fewer studies into the role of PTH signaling have been conducted. In the research presented here, *PTH1R* was found to be upregulated in the shell gland of hens at 31 weeks. Consistent with this, Thiede et al. (1991) measured *PTH1R* mRNA levels in the laying hen oviduct and found greatest expression in the shell gland. A possible role for PTH in oviduct differentiation and development as well as regulation of smooth muscle activity in this tissue was hypothesized in the aforementioned study. Furthermore, receptor binding affinity for PTH in the shell gland of laying hens (Ieda et al., 2000) and guinea fowl (Ogawa et al., 2000) was found to increase during periods of eggshell calcification, while no variations were observed in non-laying birds. This indicates a strong association of PTH with the oviposition cycle that was also evidenced by Sinclair-Black et al. (2024), where upregulation of *PTH1R* in the shell gland was observed between 21 and 24 HPOP. This correlates with findings from the present study, where tissue collection occurred at 21 HPOP and upregulation of *PTH1R* was observed. Together, this suggests that PTH signaling through PTH1R may play an important role in providing shell gland with the capacity to transfer minerals for eggshell formation.

Of the tissues examined, *CALCR* was found to only increase in kidney and shell gland after the onset of egg production, the two tissues found to have the most capacity for mineral transport in this study. Tissue collection from this study occurred when a hard-shelled egg was present in the shell gland; thus, increased *CALCR* expression in kidney and shell gland observed here are likely to ensure sensitivity to the regulatory response by calcitonin at the end of the oviposition cycle, when eggshell formation is almost complete and calcium demands for this process decrease. Yasuoka et al. (1998) demonstrated increased binding affinity of calcitonin to its receptor in the laying hen kidney between 3 h before oviposition and 2 h post-oviposition, consistent with times of lower calcium demands and when tissues were collected in the present study. Similar findings were observed in the shell gland of laying hens by Ieda et al. (2001) and guineafowl by Ogawa et al. (2003), where calcitonin binding affinity increased at later stages of eggshell formation. These results correlate with observations from Sinclair-Black et al. (2024), where mRNA levels of shell gland *CALCR* in laying hens had greater expression between 2 h before oviposition and 1 h post-oviposition. During this time, mineral requirements in the shell gland switch from high amounts of calcium to phosphorus for hydroxyapatite and cuticle deposition within the eggshell, and CALC might play an important role in this process.

Hormonal regulation of phosphorus homeostasis exerted by FGF23 signaling also appears to be upregulated in kidney and shell gland after the onset of lay, specifically observed through increased expression of *FGF* receptors and *KL*, their co-receptor. In mammals (Shimada et al., 2004b; Perwad et al., 2005) and chickens (Ren et al., 2017), FGF23 has been associated with renal excretion of excess circulating phosphorus. In the current study, renal expression of *FGFR1, FGFR4,* and *KL* was upregulated at 31 weeks. When measured throughout the 24-h oviposition cycle in Sinclair-Black et al. (2024), renal expression of *FGFR1* and *KL* had no variation, while *FGFR2*,*FGFR3*, *FGFR4* were influenced by time. In that study, a decrease in *FGFR3* was observed after 21 HPOP, so it is likely that *FGFR3* did not change here due to the time of tissue collection; however, these and other findings support the role of FGF23 signaling in this tissue to promote renal excretion of phosphorus (Kerschnitzki et al., 2014; Gloux et al., 2020a).

Findings presented here suggest that shell gland is a novel target for FGF23 signaling, as indicated by increased expression of *FGFR2 - 4* and *KL* after the onset of egg production. Signaling of FGF23 in this tissue may be associated with phosphorus uptake and utilization at the later stages of eggshell formation, as it was observed in Sinclair-Black et al. (2024) that upregulated expression of these genes occurred between 18 and 24 HPOP. Signaling by FGF23 could regulate expression of specific phosphorus transporters. Its pattern in the shell gland was followed by increased expression of *P*_*i*_ *T-1* (Sinclair-Black et al., 2024), and our findings also indicated upregulation of *P*_*i*_ *T-1, P*_*i*_ *T-2,* and *NaP*_*i*_ *llb* in the shell gland concurrently with mediators of FGF23 signaling. These changes support that FGF23 could be involved in mediating phosphorus deposition within the hydroxyapatite layer of the eggshell immediately below the cuticle that has been suggested to be required for halting calcification of the shell (Cusack et al., 2003) or formation of the cuticle itself. Taken together, these findings suggest that after the onset of lay, hens acquire greater sensitivity to hormones regulating calcium and phosphorus homeostasis in target tissues involved in mediating increased demands of these minerals for bone remodeling and eggshell formation.

Vitamin D_3_ metabolism by 25-hydroxylase, encoded by *CYP2R1* or *CYP27A1*, was found to be either downregulated or not influenced by the onset of lay in all tissues examined except kidney, which exhibited a fold increase of approximately 1.5 or higher after the onset of lay for both genes. It was only recently that *CYP27A1* was confirmed to encode for an alternative 25-hydroxylase (Shang et al., 2023), and *CYP2R1* has been catalogued as highly conserved between avian and mammalian species (Watanabe et al., 2013). Liver has been identified to be the major tissue responsible for conversion of circulating vitamin D_3_ into 25(OH)D_3_ in mammals (Ponchon et al., 1969). In avian species, early studies demonstrated *in vitro* 25-hyroxylase activity in liver homogenates (Tucker et al., 1973), as well as identified liver as an important tissue in this process through *in vivo* studies (Bhattacharyya and Deluca, 1974). However, it appears that liver is not the exclusive tissue with 25-hydroxylase activity in chickens. Tucker et al. (1973) first demonstrated that renal 25-hydroxylase from white Leghorn hens can play this role *in vitro*, which would give kidney the ability to perform the full two-step transformation of vitamin D_3_ into its active form. Similar to results presented here, Sinclair-Black et al. (2024) observed minor changes in hepatic *CYP2R1* mRNA expression over the oviposition cycle, while renal expression of this gene was noted to increase towards periods of peak eggshell calcification. Furthermore, Bhattacharyya and Deluca (1974) evaluated regulation of liver 25-hydroxylase in chickens and discussed the possibility that, although kidney may have the capacity to convert dietary vitamin D_3_ into 25(OH)D_3_, it may not necessarily contribute to circulating levels of this metabolite. This is likely associated with the fact that when synthesized in the kidney, 25(OH)D_3_ can be immediately hydroxylated into 1,25(OH)_2_D_3_ by renal 1α-hydroxylase and utilized for renal calcium reabsorption. Investigation into tissue distribution of *CYP27A1* in both male and female 8-week-old broiler chickens demonstrated an approximate 20-fold difference between liver and kidney, with liver levels being the highest amongst all tissues examined (Shang et al., 2023). This would suggest that liver remains the primary site of CYP27A1 25-hydroxylase activity, while the kidney may exhibit 25-hydroxylase activity by both enzymes for local production of 25(OH)D_3_ and, ultimately, 1,25(OH)_2_D_3_. Thus, results presented in the current study provide support for the notion that the conversion of dietary vitamin D_3_ into 25(OH)D_3_ can take place at the renal level, particularly during periods characterized by elevated calcium demands such as eggshell formation and medullary bone replenishment. Furthermore, it was observed that, although expression of genes encoding 25-hydroxylase in kidney were upregulated, circulating levels of 25(OH)D_3_ decreased at 31 weeks. This is presumably a result of greater conversion of this metabolite to 1,25(OH)_2_D_3_ in the kidney, as is evidenced by an increase in circulating 1,25(OH)_2_D_3_ and an accompanying decrease in the inactive 24,25(OH)_2_D_3_ metabolite. Collectively, activation of dietary vitamin D_3_ is upregulated by the onset of lay as a responsive mechanism to escalating calcium and phosphorus demands. Additionally, the kidney may undertake a local role in both activating hydroxylation steps that ultimately facilitate calcium reabsorption and phosphorus excretion in this tissue.

Both 25(OH)D_3_ and 1,25(OH)_2_D_3_ bind to circulating DBP to be transported to target tissues (White and Cooke, 2000). In mammals, the major synthesis site of this binding protein is the liver (Cooke et al., 1991), although expression of the gene encoding for this protein is present in several other tissues, including kidney, testis, placenta, and adipose, but it has not been detected in either intestine or uterus (McLeod and Cooke, 1989). However, to the authors’ best knowledge, no literature has been published elucidating *DBP* mRNA tissue distribution in chickens, though the protein has been detected in chicken serum (Bouillon et al., 1980). Findings from the current study confirm expression of *DBP* in laying hen kidney and liver, and unpublished data revealed levels in liver to be over 600-fold higher than those in other tissues (R.A. Garcia-Mejia, M. Sinclair-Black, and L.E. Ellestad). Similar to lack of detection in mammalian uterus, DBP was not detected in the shell gland in this study; however, it was found to be expressed in the ileum. As with 25-hydroxylase expression, kidney was found to be the only tissue where *DBP* was influenced by sexual maturation, with a 2-fold increase after the onset of lay. Research from Nys et al. (1986a) first demonstrated a marked increase in circulating DBP and 1,25(OH)_2_D_3_ in hens between 8 and 24 weeks as they approached the onset of egg production, specifically starting 2 weeks prior to the first oviposition. Further research by Nys et al. (1989) evaluated the effect of the onset of lay on plasma DBP levels by inducing sexual maturity in 12 weeks old pullets via administration of estrogen alone or in combination with testosterone, which induced an increase in DBP levels after treatment. In addition, circulating DPB levels were analyzed in mature laying hens and these were found to be even higher than those of induced immature pullets. These findings suggest a close relationship between the onset of lay and both DBP and 1,25(OH)_2_D_3_ in circulation. From the present study, the upregulation of renal *DBP* could indicate that the kidney plays a role in production of this protein following the onset of lay, which might allow this tissue to respond to 1,25(OH)_2_D_3_ more efficiently.

The expression of genes associated with calcium uptake and utilization exhibited a discernible increase following the onset of egg production, particularly in kidney, shell gland, and ileum that are all actively engaged in mineral transport. These included upregulated expression of *PMCA1* and *CALB1* in kidney, shell gland, and ileum, as well as increased *TRPV6* in the kidney. However, the calcium transporter *NCX1* was found to be downregulated in all tissues examined. Renal *NCX1* was found to be unaffected by time within the oviposition cycle in laying hens (Sinclair-Black et al., 2024) or throughout different production stages (San et al., 2021). In addition, expression of *TRPV6* in this study was only detected in the kidney, which is consistent with findings from other authors where *TRPV6* was not detected in shell gland (Sinclair-Black et al., 2024) or ileum (Proszkowiec-Weglarz and Angel, 2013). There are no reports to date for liver that the authors are aware of, though unpublished data in our lab indicated it is not detected in this tissue (R.A. Garcia-Mejia, M. Sinclair-Black, and L.E. Ellestad). In the intestine, the calcium chaperone *CALB1* has been previously reported to increase 5-fold after sexual maturity in the intestine of laying hens (Nys et al., 1992a) and 12-fold in the intestine after an intramuscular dose of 1,25(OH)_2_D_3_ (Theofan et al., 1986). The increase observed in the present study is consistent with those findings.

In the shell gland, Jonchère et al. (2012) measured ion transporter expression during the presence or absence of a calcifying egg and observed downregulation of *NCX1* and upregulation of *PMCA1* and *CALB1* during eggshell calcification, which correlates with findings from the current study. Ieda et al. (1995) reported an increase in both *VDR* and *CALB1* in the shell gland with the presence of a calcifying egg, and increased mRNA expression of *CALB1* after the onset of lay has been demonstrated in prior studies (Nys et al., 1989; Bar et al., 1990), as observed here. Furthermore, findings presented in the current study exhibited upregulation of *VDR* in shell gland following the onset of egg production, which is supported by previous research (Ieda et al., 1995; Yoshimura et al., 1997). Similarly, findings from Sinclair-Black et al. (2024) described expression of shell gland *VDR* to increase towards times of peak eggshell calcification and decline around oviposition and ovulation. However, whether shell gland *CALB1* expression is vitamin D_3_-dependent is not clear (Corradino, 1993; Yoshimura et al., 1997; Ohira et al., 1998), and it appears its expression could be driven by estrogen (Navickis et al., 1979; Corradino et al., 1993) or calcium flux (Bar et al., 1992; Nys et al., 1992b; Bar et al., 1996). Required for CaCO_3_ synthesis, expression of *CA* in the shell gland was upregulated after the onset of egg production; however, the bicarbonate transporter *SLC26A9* was found to be downregulated. This is contradictory to findings from other authors that suggest eggshell formation induces upregulation of this gene (Jonchère et al., 2012). However, the difference between results presented here and findings from the aforementioned study are likely related to the fact that tissues were collected in this study at 21 HPOP, when calcification is complete. This is supported by observations in Sinclair-Black et al. (2024), where expression of *SLC26A9* increased between ovulation and late calcification but was downregulated between 21 and 24 HPOP.

Phosphorus metabolism in laying hens is also heavily influenced by egg production, as the mechanisms required for eggshell formation involve medullary bone breakdown. The kidney has been demonstrated to play major role in regulation of calcium and phosphorus homeostasis (Wideman, 1987), and excretion of phosphorus has been shown to increase during periods of peak eggshell calcification associated with medullary bone remodeling for eggshell formation (Nys et al., 1986b; Kerschnitzki et al., 2014). In the kidney, *P*_*i*_ *T-I* appears to be the primary transporter mediating phosphorus flux during egg production, as demonstrated by a 15-fold increase in expression between 18 and 31 weeks. This finding correlates with increased renal *P*_*i*_ *T-1* and *P*_*i*_ *T-2* expression during times of peak (Sinclair-Black et al., 2024) or late (Gloux et al., 2020b) eggshell formation, probably to support renal phosphorus excretion. A similar increase was observed for shell gland *P*_*i*_ *T-I* levels; however, this tissue seemed to have an even greater increase in *NaP*_*i*_ *llb* expression, with a near 40-fold increase in at 31 weeks compared to 18 weeks. Phosphorus transport in the shell gland could increase in order to synthesize the eggshell cuticle (Sinclair-Black et al., 2024), composed of a large concentration of phosphorus (Cusack et al., 2003), as well as a thin layer of hydroxyapatite crystals beneath the cuticle, known as vertical crystal layer (Dennis et al., 1996; Kulshreshtha et al., 2022). Furthermore, as indicated previously, upregulation of phosphorus transporters in the shell gland could be mediated by FGF23 signaling. In general, hens at 31 weeks exhibited greater capacity for mineral transport, utilization, and uptake in kidney, ileum, and shell gland as calcium and phosphorus demands escalate to fulfill eggshell formation requirements.

Dietary supplementation with AlphaD3 maintained bone mineralization and breaking strength after the onset of egg production. Hens fed AlphaD3 exhibited conservation of humerus BMD and tibia breaking strength at 31 weeks, as well as higher tibia BMD and BMC at this age. Changes in bone mineralization in this study may be correlated with improved medullary bone formation in AlphaD3-fed hens compared to control-fed hens. Although medullary bone was not measured in this study and further investigation would be needed to confirm this, DEXA is considered a reliable tool to measure bone density, which is indirectly associated with medullary bone (Hester et al., 2004). The increase in BMD and BMC that was observed between 18 and 31 weeks could be attributed to changes in medullary bone, considering that cortical bone formation is limited following the onset of sexual maturity (Hudson et al., 1993; Whitehead, 2004). Given that medullary bone formation induced by the onset of sexual maturity acts as one of the major calcium reservoirs in laying hens, enhancement in this process would hold significant importance. Frost et al. (1990) reported increased tibia weight in 53-week-old laying hens after supplementation with 1α-hydroxycholecalciferol. Similar findings have been observed in broiler chickens, where increased tibia ash (Biehl et al., 1995) and strength (Han et al., 2009) were reported with 1α-hydroxycholecalciferol in the diet. Thus, our findings suggest that supplementation with AlphaD3 may facilitate medullary bone formation during early stages of the production cycle. As such, it has the potential to reduce fracture incidences as hens age, thereby countering skeletal health issues associated with high mineral demands during extended production and improving animal welfare. As AlphaD3 did not have any other major effects in this study, mechanisms by which it improved skeletal parameters are unknown but could involve direct actions on bone itself. Elucidation of AlphaD3 effects on bone could be uncovered by investigation into gene expression at the mRNA or protein level, biochemical markers for remodeling, and high-resolution structural analyses.

In conclusion, findings from this study revealed tissue-specific involvement of select transporters and hormonal signaling mechanisms potentially influencing functional changes associated with eggshell formation and bone mineralization in laying hens following the onset of egg production. The tissues evaluated exhibited a remarkable capacity to alternate between highly demanding states of increased mineral absorption, retention, or excretion in order to maintain homeostasis between eggshell formation and bone mineralization. Key regulators of mineral homeostasis in kidney and shell gland that were identified represent potential targets for strategies aimed at enhancing mineral utilization by laying hens. In addition, targeted nutritional approaches that improve efficiency of utilization and activation of dietary vitamin D_3_ could be used to optimize development of medullary bone, ultimately improving skeletal integrity and hen welfare at all stages of production.

Five Future studies that expand on the current findings should incorporate additional methods such as transcriptomics and metabolomics that allow for a more robust identification of the broader physiological pathways and processes involved. Functional validation of pathways and genes identified by analysis of protein expression and activity, as well as the use of *in vitro* methods that allow a detailed investigation into molecular and cellular processes, will ultimately provide fundamental information necessary to develop successful strategies allowing hens to efficiently utilize minerals for skeletal remodeling and eggshell deposition throughout all stages of egg production. CONFLICT OF INTEREST

The authors declare that the research was conducted in the absence of any commercial or financial relationships that could be construed as a potential conflict of interest.

## Data Availability

The raw data supporting the conclusions of this article will be made available by the authors, without undue reservation.
